# Metabolic Respiration Induces AMPK- and Ire1p-Dependent Activation of the p38-Type HOG MAPK Pathway

**DOI:** 10.1371/journal.pgen.1004734

**Published:** 2014-10-30

**Authors:** Hema Adhikari, Paul J. Cullen

**Affiliations:** Department of Biological Sciences, State University of New York at Buffalo, Buffalo, New York, United States of America; University of North Carolina at Chapel Hill, United States of America

## Abstract

Evolutionarily conserved mitogen activated protein kinase (MAPK) pathways regulate the response to stress as well as cell differentiation. In *Saccharomyces cerevisiae*, growth in non-preferred carbon sources (like galactose) induces differentiation to the filamentous cell type through an extracellular-signal regulated kinase (ERK)-type MAPK pathway. The filamentous growth MAPK pathway shares components with a p38-type High Osmolarity Glycerol response (HOG) pathway, which regulates the response to changes in osmolarity. To determine the extent of functional overlap between the MAPK pathways, comparative RNA sequencing was performed, which uncovered an unexpected role for the HOG pathway in regulating the response to growth in galactose. The HOG pathway was induced during growth in galactose, which required the nutrient regulatory AMP-dependent protein kinase (AMPK) Snf1p, an intact respiratory chain, and a functional tricarboxylic acid (TCA) cycle. The unfolded protein response (UPR) kinase Ire1p was also required for HOG pathway activation in this context. Thus, the filamentous growth and HOG pathways are both active during growth in galactose. The two pathways redundantly promoted growth in galactose, but paradoxically, they also inhibited each other's activities. Such cross-modulation was critical to optimize the differentiation response. The human fungal pathogen *Candida albicans* showed a similar regulatory circuit. Thus, an evolutionarily conserved regulatory axis links metabolic respiration and AMPK to Ire1p, which regulates a differentiation response involving the modulated activity of ERK and p38 MAPK pathways.

## Author Summary

In fungal species, differentiation to the filamentous/hyphal cell type is critical for entry into host cells and virulence. Comparative RNA sequencing was used to explore the pathways that regulate differentiation to the filamentous cell type in yeast. This approach uncovered a role for the stress-response MAPK pathway, HOG, during the increased metabolic respiration that induces filamentous growth. In this context, the AMPK Snf1p and ER stress kinase Ire1p regulated the HOG pathway. Cross-modulation between the HOG and filamentous growth (ERK-type) MAPK pathways optimized the differentiation response. The regulatory circuit described here may extend to behaviors in metazoans.

## Introduction

Organisms sense and respond to diverse stimuli through the action of signal transduction pathways. During complex behaviors like cell differentiation, multiple pathways choreograph changes in the cell cycle and cell polarity to produce a new cell type. In many cases, it is not clear what pathways are involved or how different pathways collaborate to produce major changes in cellular reprogramming. Here, we investigate the problem of cell differentiation in fungal species that differentiate to the filamentous/hyphal cell type. In pathogens, filamentous growth is critical for virulence [1], [2]. Therefore, identifying the pathways that regulate filamentous growth, and understanding how they function in an interconnected manner, is important for studies in eukaryotic cell differentiation and fungal pathogenesis.

In the budding yeast *Saccharomyces cerevisiae*, different MAPK pathways mediate the response to different stimuli. An ERK-type MAPK pathway called the filamentous growth pathway induces differentiation to the filamentous/invasive/pseudohyphal cell type in response to nutrient limitation [3], [4]. A p38-type MAPK pathway, called the high osmolarity glycerol response (HOG) pathway mediates osmoadaptation [5], [6]. The two pathways utilize some of the same components, including a core module consisting of the Rho-type GTPase Cdc42p, the p21 activated (PAK) kinase Ste20p, the MAPKKK Ste11p, and the adaptor protein Ste50p (**Fig. S1**, [5], [7]–[9] and references therein). Plasma membrane (PM) regulators of the filamentous growth pathway, Msb2p, Sho1p, and Opy2p [10]–[12], also regulate the Ste11p branch of the HOG pathway [13]–[15]. The signaling mucin Msb2p may preferentially regulate the filamentous growth pathway, as another signaling mucin, Hkr1p, has been shown to mainly regulate the HOG pathway [16], [17]. A second branch of the HOG pathway (Sln1p branch) converges on the MAPKK Pbs2p and is regulated by the two-component sensors Sln1p and Ypd1p, the protein kinase Ssk1p, and the redundant MAPKKKs Ssk2p and Ssk22p (**Fig. S1**, [13], [18]–[21]). Thus, different MAPK pathways mediate different responses through the action of common or shared signaling modules.

To date, the filamentous growth and HOG pathways are thought to control different responses [22]–[25]. The filamentous growth pathway induces differentiation into chains of branched interconnected filaments by regulating changes in the cell cycle [26]–[28], cell adhesion [29], [30], and cell polarity [31]–[33]. By comparison, the HOG pathway induces transient growth arrest [6], [34], [35] by the phosphorylation of translation initiation factors [36]–[38]. The HOG pathway also controls the production of osmolyte ([39] and references therein) and regulates changes in chromatin architecture [40]–[43], yet does not trigger a morphogenetic response. In fact, osmotic stress transiently depolarizes the actin cytoskeleton [44]–[46]. Further evidence that the pathways operate separate programs comes from the fact that the HOG pathway shuts off the filamentous growth pathway in response to osmotic shock [16], [47]–[49]. The filamentous growth pathway can likewise attenuate the HOG pathway [10].

The paradigm that signaling pathways (even ones that share components) function as separate entities is complicated by the fact that under certain conditions the concerted action of multiple MAPK pathways occurs. The HOG and protein kinase C (PKC, [50]) pathways are together required in some settings [51], where they act cooperatively [52] or sequentially [53]. During mating [54], [55], the PKC pathway maintains cell integrity [56], [57], and the HOG pathway balances changes in osmolarity [35]. Severe stress, like enzymatic digestion of the cell wall [58], [59] or defects in protein glycosylation [60] trigger multiple pathways as part of an ill-defined response. Diverse stimuli also trigger a general stress response, called the environmental stress response (ESR, [61], [62]) but how this is related to MAPK-dependent responses is not clear.

Here, comparative RNA sequencing (RNA seq) analysis was used to examine the transcriptional response to inducers of the HOG (salt) and filamentous growth (galactose) pathways. The response to glycosylation deficiency was also examined, because that stress requires the action of both pathways. Analysis of expression profiling data uncovered galactose as an inducer of the HOG pathway. Accordingly, the AMPK Snf1p and an intact TCA cycle were required to activate the HOG pathway during growth on galactose. The HOG pathway has recently been shown to be activated in response to ER stress and require the unfolded protein response (UPR) regulator Ire1p [63], [64]. Growth of cells in galactose induced Ire1p-dependent activation of the HOG pathway. Therefore, the HOG and filamentous growth pathways are both induced during growth on galactose. Both pathways contributed to growth under this condition, and remarkably, both pathways attenuated each other's activities. Modulation of the filamentous growth pathway by the HOG pathway was required to optimize the differentiation response. The regulatory circuit described here connects general regulators of cellular responses – AMPK, Ire1p, and MAPK (p38 and ERK) – in a regulatory axis that controls cell differentiation. This axis was conserved in other fungal species and may underlie differentiation-type responses in metazoans, which contain evolutionarily conserved regulatory pathways.

## Results

### Expression Profiling by RNA Seq Uncovers a Role for the HOG Pathway during Growth in Galactose

**Figure 1:**
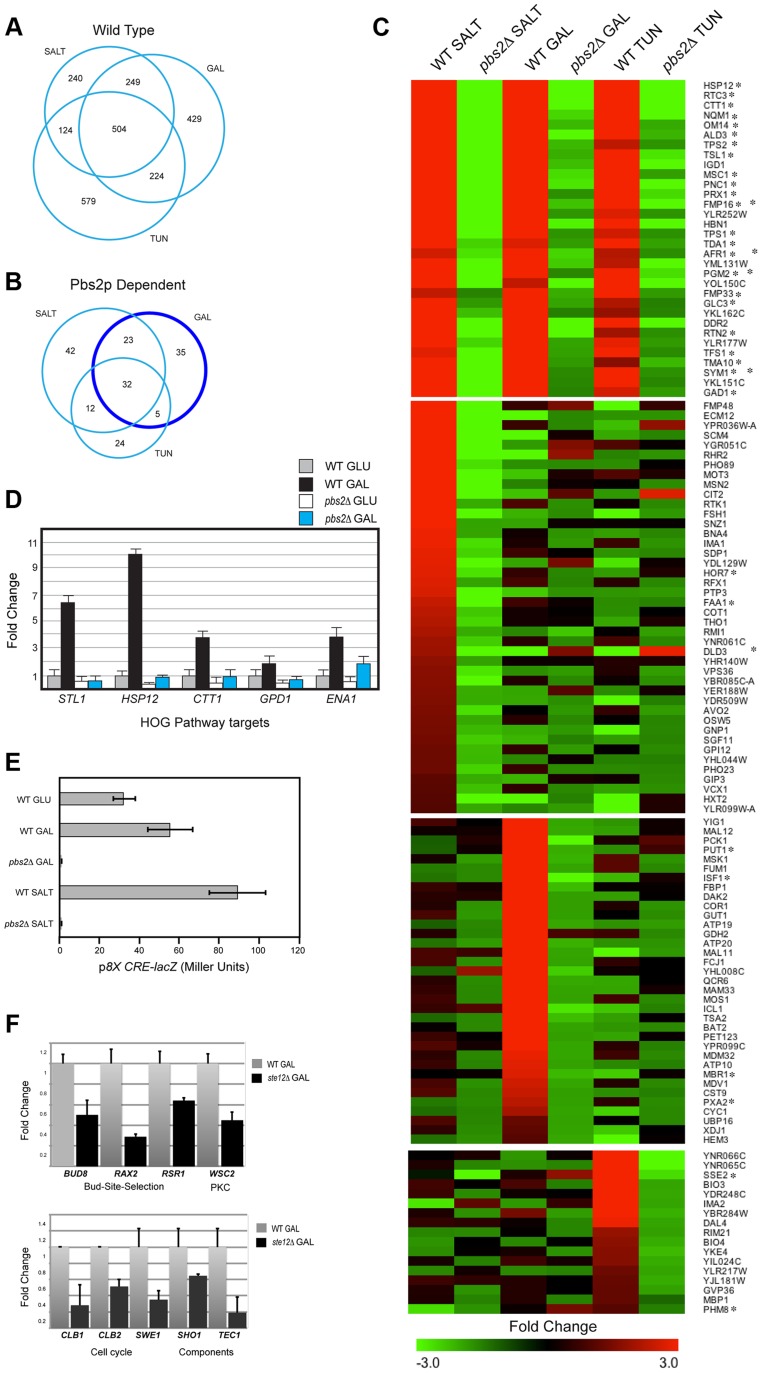
Gene expression profiling by RNA seq analysis and qPCR. **A**) Genes induced by salt, tunicamycin (TUN), or galactose (GAL). All RNA seq comparisons are provided in Table S1. **B**) Genes induced in a Pbs2p-dependent manner under the indicated conditions. Genes outlined by the dark blue circle (Pbs2p-dependent GAL specific) were functionally annotated in a pie chart in **Fig. S2**. **C**) Heat map of genes induced by the indicated stresses. Common targets and targets unique to each stimulus is shown. Asterisk, target of ESR. **D**) qPCR of HOG pathway target mRNAs in wild type and the *pbs2*Δ mutant grown in glucose (GLU, YEPD) and galactose (GAL, YEP-GAL). Error bars indicate +/−S.E.M. of three independent experiments. Actin (*ACT1*) mRNA was used as a control. **E**) Activity of p8X*CRE-lacZ* in wild-type cells (PC313) and *pbs2*Δ mutant (PC5035) grown in YEPD (5.5 hr), YEP-GAL (5.5 hr), and YEPD+0.4 M KCl (30 min). **F**) qPCR of Ste12p target mRNAs in wild type (PC538) and the *ste12*Δ (PC2382) mutant grown in glucose (YEPD) and galactose (YEP-GAL). See panel D for details.

Comparative RNA sequencing (RNA seq, [65]) was performed to examine the response of *S. cerevisiae* cells to different stimuli. The response to osmotic stress (YEPD+0.4M KCl [23]), the non-preferred carbon source galactose (YEP-GAL, 2% GAL [66]), and an inhibitor of N-linked glycosylation (YEPD+2.5 µg tunicamycin [67]) were examined. Each stimulus induced the expression of overlapping and non-overlapping genes (**Fig. 1A**). As reported, salt induced targets of the HOG pathway (Table S1, [12], [24]), galactose induced the *GAL* genes and other starvation-responsive genes (Table S1, [68], [69]), and tunicamycin induced targets of the UPR and other genes (Table S1, [70], [71]). The different stimuli also induced an overlapping gene set (**Fig. 1A**, 504 genes). Common genes included targets of the ESR (150 of 504 total, Table S1 [61], [62]) and targets of the HOG pathway. Likewise, a partially overlapping set of repressed genes was also identified that included ESR targets (Fig. S2A).

To explore the role of the HOG pathway in response to different stresses, RNA seq profiles were compared between wild-type cells and cells lacking the HOG pathway MAPKK Pbs2p [*pbs2*Δ]. An overlapping set of Pbs2p-dependent genes was induced in response to all three conditions (32 genes, **Fig. 1, B and C**). Many of the genes (**Fig. 1C**, 27/32, asterisks) are targets of the ESR. The HOG response to the different stimuli was complex: Pbs2p-dependent targets showed different levels of induction by the different inducers and different induction profiles. For example, different subsets of Pbs2p-dependent targets were unique to each stimulus (**Fig. 1, B and C**; Table S1) or common to two of the three stimuli (**Fig. 1B**). Thus, the HOG pathway actuates common and unique outputs in response to different stimuli.

The finding that galactose induced HOG pathway targets in a Pbs2p-dependent manner was unexpected. Pbs2p-dependent targets of the HOG pathway that were induced during growth in galactose regulate mitochondria/respiration, carbohydrate metabolism (including gluconeogenesis, glyoxylate cycle, glycogen metabolism), and amino acid/nitrogen metabolism (Fig. S2B; Table S1). Quantitative PCR (qPCR) analysis confirmed galactose- and Pbs2p-dependent induction of HOG targets, which included established targets of the HOG pathway (*STL1*, *ENA1*, *GPD1*, *CTT1*, and *HSP12*) to varying levels corresponding to the RNA seq analysis (**Fig. 1D**, [23], [24], [41], [72]). Likewise, the HOG pathway reporter p*8XCRE-lacZ* [73] was induced by galactose (by 2.3-fold) in a Pbs2p-dependent manner (**Fig. 1E**; salt induced the reporter by 3-fold). Therefore, the HOG pathway induces a transcriptional response during growth in galactose.

The filamentous growth pathway is induced during growth in galactose [16], [74], and cells undergo filamentous growth in this setting. The filamentous growth pathway shares components with the HOG pathway. Comparative RNA seq between wild-type cells and the *ste12*Δ mutant showed that the HOG and filamentous growth pathways induce different target genes. Known targets of the filamentous growth pathway were identified (*FLO11*, *CLN1*, *PGU1*, *YLRO42C*, *BAR1*, *MSB2*, Table S1, [11], [22], [27]) as well as new targets, including genes that regulate progression through the G_2_/M phase of the cell cycle (*CLB1*, *CLB2* and *SWE1*, [75], [76]; **Fig. 1F**), bud-site-selection (**Fig. 1F**, *BUD8*, *RAX2* and *RSR1*, [77]–[79]), a PM regulator of the PKC pathway (**Fig. 1F**, *WSC2* [80]), and components of the filamentous growth pathway (**Fig. 1F**, *SHO1* and *TEC1* [12], [23], [81]), possibly leading to positive feedback [82]. These genes were not Pbs2p-dependent, and the filamentous growth pathway did not show induction of HOG pathway targets (Table S1). Therefore, the HOG and filamentous growth pathways mediate non-overlapping responses during growth on galactose.

### Comparing HOG Pathway Activation by Galactose and Osmotic Stress

**Figure 2:**
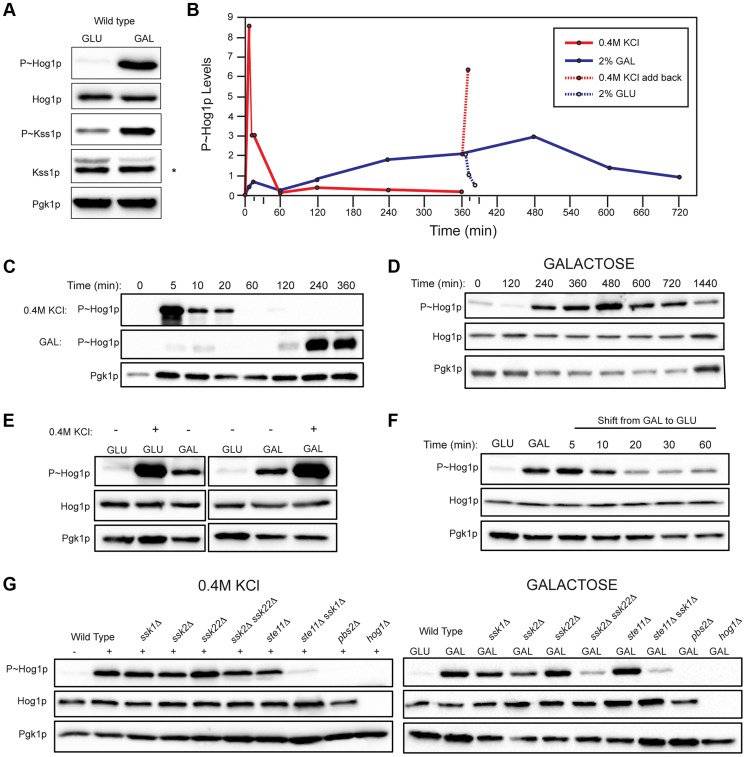
Comparison of HOG pathway activation by galactose and osmotic stress. For all phosphoblots involving Hog1p and Kss1p, the sizes of proteins are P∼Hog1p (∼49 kDa), Hog1p (∼49 kDa), P∼Kss1p (∼43 kDa), Kss1p (∼43 kDa), and Pgk1p (∼45 kDa). Pgk1p was used as a loading control. Asterisk (*) refers to a background band detected by the Kss1p antibody. Basal P∼Hog1p and P∼Kss1p showed variable levels under un-inducing conditions. **A**) Wild type cells (PC538) cells were grown to mid-log phase (∼5.5 hrs) in YEPD (GLU) or YEP-GAL (GAL) media and evaluated by immunoblot analysis for phosphorylation of the MAPKs Hog1p and Kss1p. **B**) Graph of P∼Hog1p levels under the indicated conditions, as determined by ImageJ analysis. **C**) Time-course analysis. Wild-type cells (PC538) were grown to mid-log phase and transferred to media containing salt (YEPD+0.4 M KCl) or galactose (YEP-GAL) for the indicated times. **D**) Extended time course of Hog1∼P during growth in galactose. **E**) Combinatorial analysis of the response to osmotic stress and galactose. Cells were grown to mid-log phase in YEPD, YEP-GAL, or YEPD+0.4M KCl, which was added to the cells growing in YEPD for 5 min. **F**) P∼Hog1p levels in cells shifted from galactose (YEP-GAL) to glucose (YEPD) for the indicated time points. Cells in YEP-GAL media were harvested by centrifugation, washed twice in water, and resuspended in YEPD for the indicated time points. **G**) P∼Hog1p levels during growth in 0.4M KCl and galactose in mutants lacking Ssk1p or Ste11p branches of the HOG pathway. Wild type cells (PC538), and the *ssk1*Δ (PC1523), *ssk2*Δ (PC6086), *ssk22*Δ (PC6085), *ssk2*Δ *ssk22*Δ (PC6031), *ste11*Δ (PC3861), *ste11*Δ *ssk1*Δ (PC2061), *pbs2*Δ (PC2053) and *hog1*Δ (PC6047) mutants were grown in YEP-GAL medium or YEPD medium containing 0.4M KCl for 5 min.

To further examine HOG pathway activation during growth in galactose, phosphorylation of the MAPK Hog1p (P∼Hog1p) was measured, which provides a readout of HOG pathway activity [73], [83]. This assay had the advantage of evaluating the kinetics and genetic pathways required for pathway activation. Consistent with the RNA seq analysis, growth of cells in galactose induced the activation of the HOG (**Fig. 2A**, P∼Hog1p) and filamentous growth pathways (**Fig. 2A**, P∼Kss1p). Depending on the condition, different levels of basal P∼Hog1p and P∼Kss1p were detected, which may represent differences in baseline activity between the pathways.

Other nutrient-limiting conditions were also tested. Non-fermentable carbon sources, acetate and ethanol-glycerol (**Fig. S3A**), and limitation of environmental nitrogen (**Fig. S3B**), also activated the HOG pathway. Other poor nutrients, like raffinose (**Fig. S3C**), limiting glucose (**Fig. S3C**), and phosphate limitation (**Fig. S3D**) did not induce the HOG pathway, which indicates that a particular metabolic context evokes the response (see below). Because galactose caused robust induction of the HOG and filamentous growth pathways, that inducer was used for subsequent experiments.

An established trigger of the HOG pathway is an increase in external osmolarity [84]. HOG pathway activation by high osmolarity (KCl, 0.4 M) and galactose (2%) was compared. In response to osmotic stress, P∼Hog1p was detected by 5 min. By 60 min, P∼Hog1p was reduced due to pathway attenuation (**Fig. 2, B and C**, upper panel [84], [85]). By comparison, P∼Hog1p was detected during growth in galactose at 240 min (**Fig. 2, B and C**, middle panel), and the signal persisted until 720 min (**Fig. 2D**). Salt (0.4 M KCl) induced higher levels of P∼Hog1p than growth in galactose (**Fig. 2E**), which is consistent with the RNA seq analysis (Table S1). Salt (0.4 M KCl) added to cells grown in galactose caused a rapid increase in P∼Hog1p (**Fig. 2, B and E**). Transfer of cells from galactose (YEP-GAL) to glucose (YEPD), which leads to glucose repression (see below), caused a reduction in P∼Hog1p levels by 20 min **(** **Fig. 2, B and F**), which was comparable to the reduction in P∼Hog1p levels in response to osmotic shock (**Fig. 2, B and C**). Therefore, the amplitude and duration of HOG pathway signaling differs depending on whether the inducer is osmotic stress or galactose.

Two branches of the HOG pathway regulate the response to osmotic stress, which converge on the MAPKK Pbs2p (**Fig. S1**, [13], [18], [19]). In response to salt, the branches are redundant, in that mutants lacking both branches (*ssk1*Δ *ste11*Δ) show the same defect as mutants lacking the MAPKK (*pbs2*Δ) or MAPK (*hog1*Δ, **Fig. 2G**). During growth in galactose, but not salt, the MAPKKKs Ssk2p and Ssk22p were required for HOG pathway activation (*ssk2*Δ *ssk22*Δ; **Fig. 2G**). Thus, Ssk2p and Ssk22p have a role in the response to galactose that differs from their role in response to osmotic stress. The different branches of the HOG pathway play different roles under different conditions, such as in the response to different concentrations of salt [23]. Nitrogen limitation showed a similar requirement for Ssk2p and Ssk22p (**Fig. S3E**). The Ste11p branch alone was not required (*ste11*Δ, **Fig. 2G**), but when the *ste11*Δ mutant was combined with the *ssk1*Δ mutant, a defect was observed (*ssk1*Δ *ste11*Δ, **Fig. 2G**). Thus, the Ste11p branch plays a minor role in the HOG response to galactose. One might expect that the Msb2p/Sho1p branch, which induces the filamentous growth pathway in response nutrient limitation, would transmit nutrient signals to Pbs2p/Hog1p (**Fig. S1**). In fact, the Sln1p branch played the major role in this nutritional response, whereas the Sho1p branch played a minor role.

### The AMPK Snf1p and the TCA Cycle Are Required for HOG Pathway Activation by Galactose

**Figure 3:**
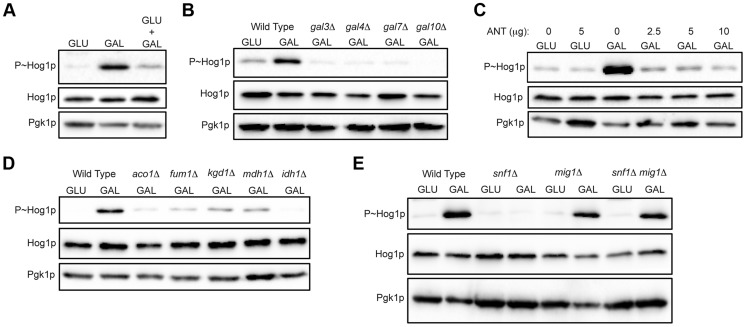
Role of increased metabolic respiration and Snf1p in activation of the HOG pathway. **A**) Immunoblot showing P∼Hog1p levels in cells grown in glucose (YEPD), galactose (YEP-GAL) or glucose and galactose (YEPD+2% GAL). **B**) Wild-type cells (PC6016) and the *gal3*Δ, *gal4*Δ, *gal7*Δ and *gal10*Δ mutants grown in YEP-GAL. **C**) P∼Hog1 levels in cells grown under the indicated conditions for 3 h with or without antimycin, ANT. **D**) Wild type (PC538) and the *aco1*Δ (PC3912), *fum1*Δ (PC6152), *mdh1*Δ (PC6153) and *kgd1*Δ (PC6155) and *idh1*Δ (PC6154) mutants were grown in galactose for 5.5 hrs. **E**) Wild-type cells (PC538), and the *snf1*Δ (PC560), *mig1*Δ (PC4843) and *snf1*Δ *mig1*Δ (PC6076) mutants were grown in YEP-GAL medium for 5.5 hrs.

Glucose is the preferred carbon source in yeast [86], [87]. When glucose is abundant, yeast cells exclusively utilize that nutrient over non-preferred carbon sources like galactose. Glucose repression prevents the transport and utilization of other carbon sources [86], [88]–[90]. As shown above, glucose added to cells grown on galactose resulted in attenuation of the HOG response (**Fig. 2F**). To further test whether glucose prevents the HOG response to galactose, cells were grown in media containing both glucose and galactose as a carbon source. Under this condition, HOG pathway signaling was also attenuated (**Fig. 3A**). These experiments indicate that galactose metabolism is required for HOG pathway activation. Consistent with this possibility, mutants defective for galactose transport and utilization (**Fig. 3B**; *gal3*Δ, *gal4*Δ, *gal7*Δ, and *gal10*Δ [88], [90]–[93]) were defective for HOG pathway activation.

Galactose utilization increases the respiratory capacity by shunting ATP production through the electron transport chain [86], [94], [95]. An inhibitor of respiration, antimycin [96]–[98], prevented HOG pathway activation by galactose (ANT, **Fig. 3C**). Antimycin did not prevent HOG pathway activation in response to salt (**Fig. S4A**). Metabolic respiration produces intermediates that are utilized by the tricarboxylic acid (TCA, or citric acid) cycle to generate precursors for electron transport. Strains lacking TCA cycle enzymes aconitase (*aco1*Δ), fumarase (*fum1*Δ), malate dehydrogenase (*mdh1*Δ), alpha keto-glutarate (*kgd1*Δ) and iso-citrate dehydrogenase (*idh1*Δ) were defective for HOG pathway activation by galactose (**Fig. 3D**). These mutants were not required to mediate an osmotic response (**Fig. S4B**, shown for *aco1*Δ). Therefore, metabolic respiration of galactose underlies activation of the HOG pathway.

The AMP-dependent protein kinase (AMPK) Snf1p is a major regulator of the response to poor carbon source utilization [99], [100]. The main function of Snf1p is the de-repression of glucose-repressed genes [86], [89], [99]. Snf1p was required for HOG pathway activation by galactose (**Fig. 3E**). Snf1p functions with the regulatory subunit Snf4p [101], which was also required for HOG pathway activation by galactose (**Fig. S4C**). Snf1p and Snf4p were not required for HOG pathway activation in response to osmotic stress (**Fig. S4D**). Snf1p phosphorylates the transcriptional repressor Mig1p to relieve glucose repression [102]–[104], which leads to induction of the *GAL* genes and other genes [89], [102], [105], [106]. Loss of Mig1p restored HOG pathway activity in the *snf1*Δ mutant **(** **Fig. 3E**, *mig1*Δ *snf1*Δ). Cells lacking Mig1p alone did not influence HOG pathway activity (**Fig. 3E**, *mig1*Δ). Therefore, Snf1p regulates the HOG pathway through its major role in relieving de-repression of glucose-repressed genes. Snf1p also regulates nitrogen assimilation pathways [107] but was not required to activate the HOG pathway in response to nitrogen deficiency (**Fig. S4E**). In summary, metabolic respiration triggers AMPK-dependent activation of the HOG pathway.

### UPR Kinase Ire1p Mediates HOG Pathway Activation by Galactose

**Figure 4:**
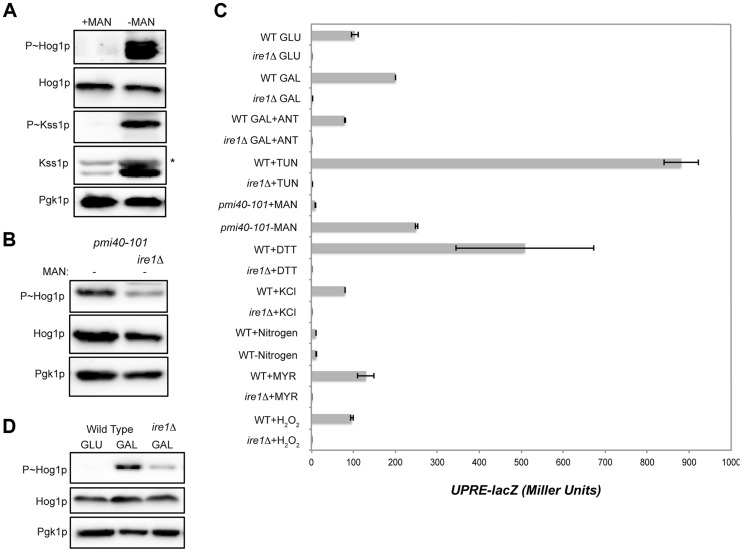
Role of the UPR in mediating HOG pathway activation during growth in galactose and in response to protein glycosylation deficiency. **A**) P∼Hog1p and P∼Kss1p levels in the *pmi40-101* mutant (PC244) grown in YEPD+/−50 mM MAN (mannose) for 5.5 hrs. **B**) The *pmi40-101* (PC244) and *pmi40-101 ire1*Δ (PC6044) mutants were grown in YEPD medium +/−50 mM MAN. **C**) Activity of the *UPRE-lacZ* reporter under the indicated conditions. H_2_O_2_ (5 mM; 3 hr), DTT (4 mM; 3 hr), KCl (1M; 30 min), TUN (2.5 µg; 3 hr), galactose (2%, 5.5 hr), MYR (myriocin) (5 µg; 3 hr), SD (+/−nitrogen; 5.5 hr), *pmi40-101* (+/− MAN; 5.5 hr), ANT (2.5 µg; 3 hr). **D**) P∼Hog1p levels in the *ire1*Δ mutant grown in galactose. Wild-type cells (PC538) and the *ire1*Δ mutant (PC6048) were grown in YEP-GAL medium.

Protein glycosylation is an oligosaccharide modification of proteins that occurs in the endoplasmic reticulum (ER) and Golgi apparatus [108]. Defects in protein glycosylation trigger a global response that involves the action of several MAPK pathways, including the filamentous growth [10], [60] and HOG pathways [63], [64]. Comparative RNA seq analysis identified HOG pathway targets induced by treatment with tunicamycin, an inhibitor of N-linked glycosylation (**Fig. 1, B and C**; Table S1). To further explore the HOG and filamentous growth pathway response to glycosylation deficiency, a conditional mutant, *pmi40-101* [60], was used that is defective for an early step in N- and O-linked glycosylation [109], [110]. Growth of the *pmi40-101* mutant in media lacking mannose induces its glycosylation defect and showed elevated HOG and filamentous growth pathway activity (**Fig. 4A**). Defects in O-linked glycosylation also modestly activated the HOG pathway (**Fig. S5A**). In response to glycosylation deficiency, HOG pathway activation did not require Snf1p (**Fig. S5B**), which is consistent with the idea that Snf1p regulates the HOG pathway by the de-repression of glucose-repressed genes.

Defects in protein glycosylation induce problems with protein folding in the ER, which activates the UPR [111], [112]. The UPR is regulated by the kinase Ire1p [113], which has recently been shown to mediate the HOG pathway response to glycosylation deficiency (**Fig. 4B** [63], [64]). As expected from these reports, Ire1p was required to mediate the HOG response to glycosylation deficiency (**Fig. 4B**). Protein glycosylation deficiency, as induced by tunicamycin or the *pmi40-101* mutant, also induced a transcriptional reporter of the UPR (*UPRE-lacZ*, [113]) in an Ire1p-dependent manner (**Fig. 4C**).

An increase in metabolic respiration might also trigger ER stress that leads to Ire1p-dependent activation of the HOG pathway. HOG pathway activation in response to galactose was reduced in the *ire1*Δ mutant (**Fig. 4D**). Galactose also induced expression of the *UPRE-lacZ* reporter in an Ire1p-dependent manner (**Fig. 4C**). Induction of the *UPRE-lacZ* reporter by galactose was abolished by treatment with antimycin (**Fig. 4C**). Therefore, an increase in metabolic respiration stimulates the UPR, which leads to Ire1p-dependent activation of the HOG pathway.

The HOG pathway is activated by several stimuli, including salt, nitrogen (this study), myriocin [14], [114], and oxidative stress [115], [116]. The UPR was not induced by these stimuli (**Fig. 4C**). Therefore, two different types of inducers activate the HOG pathway. One type is Ire1p-dependent (induced by increased metabolic respiration and glycosylation deficiency), and another is Ire1p-independent (induced by salt and other stresses).

### Filamentous Growth and HOG Pathways Contribute to Growth in Galactose and Modulate Each Other's Activities to Produce an Optimal Response

**Figure 5:**
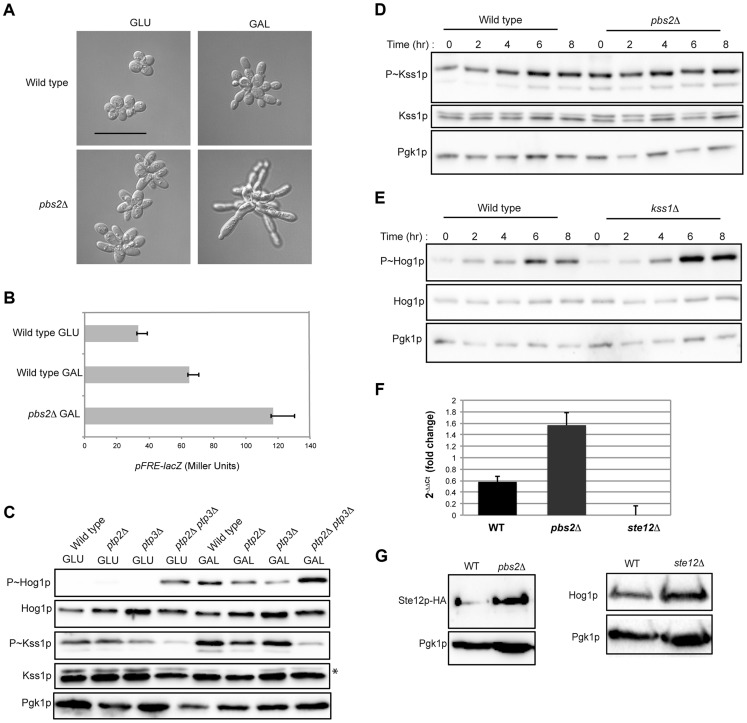
Cross-inhibition between the filamentous growth and HOG pathways during growth in galactose. **A**) Morphology of wild-type cells (PC538) and the *pbs2*Δ mutant (PC2053), grown on YEPD and YEP-GAL for 24 hrs. Bar, 5 microns. **B**) p*FRE-lacZ* reporter activity in wild-type cells (PC313) and the *pbs2*Δ mutant (PC5035) in YEP-GAL medium. **C**) Role of protein tyrosine phosphatases in P∼Hog1p activity in galactose. Wild-type cells (PC538), and the *ptp2*Δ (PC6156), *ptp3*Δ (PC6157) and *ptp2*Δ *ptp3*Δ double mutant (PC6158) were grown in YEPD and YEP-GAL media for 5.5 hrs. **D**) P∼Kss1p activity in wild-type cells and the *pbs2*Δ mutant (PC2053) grown in YEP-GAL medium over a time course as indicated. **E**) P∼Hog1p activity in the *kss1*Δ mutant (PC620) grown in YEP-GAL medium for the times indicated. **F**) qPCR showing the relative expression of *STE12* mRNA in the wild-type (PC538), *pbs2*Δ (PC2053) and *ste12*Δ (PC2382) mutant cells. Error bars indicate +/− standard error mean of three independent experiments. Actin (*ACT1*) mRNA was used as a control. **G**) Ste12p-HA protein levels in the wild-type and *pbs2*Δ strains. Hog1p levels by immunoblot analysis are also shown.

The filamentous growth and HOG pathways are activated during growth in galactose (**Fig. 1A** **;** **Fig. 2A**; Table S1). However, the HOG pathway inhibits the filamentous growth pathway in response to osmotic stress [12], [16], [47]–[49]. To determine whether the HOG pathway inhibits the filamentous growth pathway during growth in galactose, cells were examined by microscopy. Cells lacking an intact HOG pathway showed hyper-polarized growth (**Fig. 5A**, *pbs2*Δ), indicative of a hyperactive filamentous growth pathway. Comparative RNA seq showed that transcriptional targets of the filamentous growth pathway (*PGU1*, *SVS1*, *MSB2*, and *KSS1*; Table S1) were up-regulated in the *pbs2*Δ mutant in galactose. In line with the RNA seq data, the *pbs2*Δ mutant showed elevated activity of a filamentous growth pathway reporter **(** **Fig. 5B**, *FRE-lacZ*). Tyrosine phosphatases Ptp2p and Ptp3 negatively regulate the Hog1p pathway [85], [117]. The *ptp2*Δ *ptp3*Δ double mutant showed elevated P∼Hog1p levels and correspondingly lower levels of P∼Kss1p under pathway inducing conditions (galactose) (**Fig. 5C**). This was also observed under basal conditions (glucose) (**Fig. 5C**). Accordingly, the *ptp2*Δ *ptp3*Δ double mutant (and the *ptp3*Δ single mutant) showed reduced invasive growth and crosstalk reporter activity (**Fig. S6**). Therefore, the HOG pathway inhibits the filamentous growth pathway during growth in galactose.

**Figure 6:**
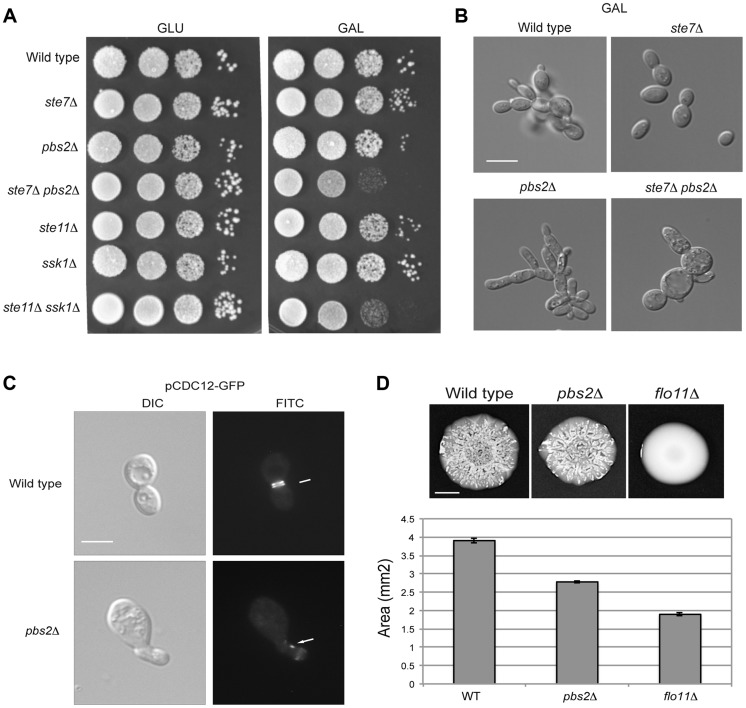
Role of the HOG and filamentous growth pathways in growth in galactose and effect of the inhibitory role of the HOG pathway on filamentous growth pathway outputs. **A**) Serial dilutions of wild-type (PC313), *ste7*Δ (PC4928), *pbs2*Δ (PC5035) and *ste7*Δ *pbs2*Δ (PC6272) cells were spotted on YEPD and YEP-GAL media. **B**) Morphology of wild-type cells (PC538), the *pbs2*Δ mutant (PC2053), the *ste7*Δ mutant (PC4982), and the *ste7*Δ *pbs2*Δ double mutant (PC6272) grown YEP-GAL media for 24 hrs. Bar, 5 microns. **C**) Septin staining of wild-type and *pbs2*Δ cells harboring the pCdc12p-GFP plasmid. Cells were grown to mid-log phase in YEPD. **D**) Mat formation in cells lacking the filamentous growth or HOG pathways. Wild-type (PC538), *flo11*Δ (PC1029), and *pbs2*Δ (PC2053) strains were grown in YEPD medium for 16 hrs and then spotted onto low agar (0.3%) YEP-GAL medium for 3 d at 30°C. Bar, 1 cm.

The filamentous growth pathway also inhibits the HOG pathway [10]. Consistent with this finding, the level of P∼Kss1p was elevated in the *pbs2*Δ mutant at 0 h, 2 h, and 4 h (**Fig. 5D**) of growth in galactose. Similarly, the level of P∼Hog1p was elevated in the *kss1*Δ mutant at 6 h and 8 h (**Fig. 5E**). RNA seq analysis showed *STE12* was up-regulated by galactose in the *pbs2*Δ mutant (Table S1). This was confirmed by qPCR (1.52 log_2_ fold) (**Fig. 5F**) and was reflected at the protein level (**Fig. 5G**). Likewise, Hog1p protein levels were modestly affected in the *ste12*Δ mutant (**Fig. 5G**). Thus, the HOG pathway inhibits the filamentous growth pathway during growth in galactose, which may occur at the *STE12* level. These results show that the HOG and filamentous growth pathways modulate each other's activities in a complex pattern in galactose.To this point, our results suggest an apparent paradox. The HOG and filamentous growth pathways are both activated during growth on galactose, yet the pathways dampen each other's activities. To determine the roles of the pathways in this setting, mutants in the filamentous growth and HOG pathway pathways were examined for growth in galactose. Mutants lacking the filamentation MAPKK (*ste7*Δ) and HOG MAPKK (*pbs2*Δ) were not defective for growth on galactose (**Fig. 6A**). However, the *ste7*Δ *pbs2*Δ double mutant showed a growth defect (**Fig. 6A**). This defect was not specific for the MAPKKs, because another mutant that blocks the activity of both pathways showed an equivalent growth defect (*ste11*Δ *ssk1*Δ, **Fig. 6A**). The *ste7*Δ *pbs2*Δ double mutant also showed morphological defects during growth in galactose (**Fig. 6B**). Therefore, the HOG and filamentous growth pathways have a redundant function in promoting proper growth and morphogenesis during growth in galactose.

What is the benefit to the cross-modulation between the two pathways? One possibility is that modulation of the pathways' activities may be important to optimize the response. To test this possibility, the *pbs2*Δ mutant was examined in detail by microscopic examination. A subset of *pbs2*Δ cells showed morphological defects [∼5% compared to <0.5% of wild-type cells, >1000 cells counted (**Fig. 6C**)]. Thus, hyper-activation of the filamentous growth pathway can lead to morphogenetic defects. To further explore this possibility, the pattern of septins, which mark the mother-bud neck [118], [119], was also examined. Septin staining showed an irregular pattern *pbs2*Δ cells with morphological defects (**Fig. 6C**). This defect is indicative of problems with cell-cycle progression or proper growth.

To determine if the hyper-polarized growth of the *pbs2*Δ mutant comes from hyper-activation of the filamentous growth pathway, cell morphology was quantitated by microscopy. In rich media (YEPD), wild-type cells grow predominately in the vegetative (round) form (8+/−2% elongated cells; 200 cells counted for all trials). By comparison, the *pbs2*Δ mutant shows a cell-elongation morphology (>83.5+/−5% elongated cells) that was abolished in the *pbs2*Δ *ste7*Δ double mutant (7+/−3% elongated cells). Therefore, the enhanced polarized morphology seen in *pbs2*Δ cells, and concomitant morphological abnormalities, is due to Ste7p. This result is complicated because in YEP-GAL, the *ste7*Δ *pbs2*Δ double mutant shows morphological defects not seen in either single mutant (**Fig. 6B**). Thus, Pbs2p may attenuate morphogenesis in multiple ways.

This finding extends to other negative-regulatory inputs to the filamentous growth pathway as well (Chavel *et al.* IN PRESS). Although only a low percentage of cells exhibit morphological defects, it is likely that even minor mis-coordination of basic cellular processes would be detrimental to cell health. Thus, modulation of the filamentous growth pathway by the HOG pathway is necessary for proper cell growth and morphogenesis.

As a second test, the response of a population of cells was examined. Yeast cells expand in biofilms/mats through the action of the filamentous growth pathway, which regulates expression of the cell-adhesion molecule Flo11p [120]. When hyper-activated, the filamentous growth pathway causes an increase in *FLO11* expression that prevents biofilm/mat expansion [121], [122]. We found that the *pbs2*Δ mutant formed smaller biofilms/mats than wild-type cells during expansion on galactose media (**Fig. 6D**, see quantitation in graph). Thus, modulation of the filamentous growth pathway by the HOG pathway is required to coordinate cell growth and optimize colonial behavioral responses.

### The Ire1p-HOG1p-ERK-p38 Axis Is an Evolutionarily Conserved Response among Fungal Species

**Figure 7:**
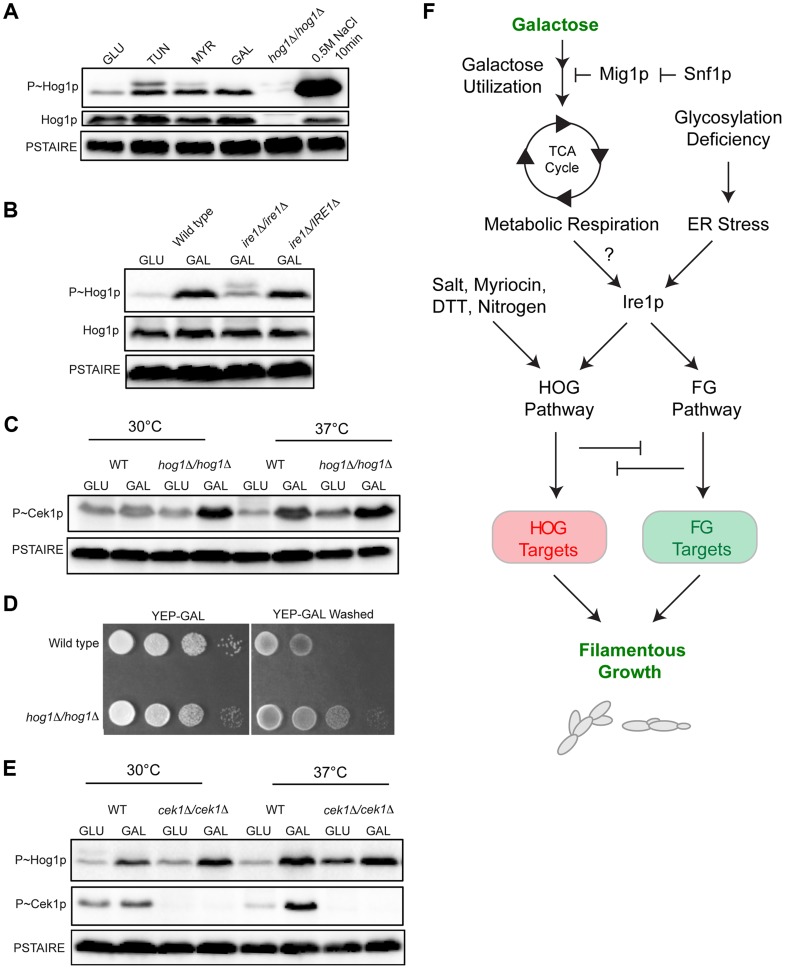
MAPK responses in *C. albicans* during growth in galactose. **A**) Immunoblot analysis of P∼CaHog1p. Wild-type (PC6111) cells were grown in YEPD and YEP-GAL medium (5.5 hrs) and treated with TUN (tunicamycin) (2.5 µg for 3 hrs), MYR (myriocin) (2.5 µg for 3 hrs) and 0.5M NaCl (10 min). **B**) Phosphorylation of CaHog1p requires the CaIre1p. Wild-type (PC6116), *ire1*Δ/*ire1*Δ (PC6144), and *ire1*Δ/*pIRE1* (PC6145) cells were grown in YEPD and YEP-GAL medium (5.5 hrs). **C**) P∼Cek1p levels in the *hog1*Δ/*hog1*Δ mutant at 30°C and 37°C. Wild-type (PC6111) and *hog1*Δ/*hog1*Δ (PC5008) cells were grown in YEPD and YEP-GAL medium (5.5 hrs). **D**) Plate-washing assay of wild-type cells (PC6111) and the *hog1*Δ/*hog1*Δ mutant (PC5008) on YEP-GAL medium at 37°C for 48 hrs. The plate was photographed, washed, and photographed again to reveal invaded cells. **E**) P∼CaHog1p and P∼Cek1p levels in the *cek1*Δ/*cek1*Δ mutant at 30°C and 37°C. Wild-type (PC6111) and *cek1*Δ/*cek1*Δ (PC6114) cells were grown in YEPD and YEP-GAL medium (5.5 hrs). **F**) Model showing the roles of the HOG and filamentous growth pathways in the response to growth in galactose. Galactose is transported into cells and metabolized by genes under the control of Snf1p. As a result, metabolic respiration is increased, which by some mechanism (?) induces the UPR. Ire1p mediates activation of the HOG and filamentous growth pathways (Adhikari et al. SUBMITTED). The HOG and filamentous growth pathways induce different target genes to redundantly promote growth under this condition. The antagonistic roles of these pathways on each other's activities optimize the response.

The signaling circuit characterized here might be specific to *S. cerevisiae* or extend to other species. To address this question, pathways of the fungal pathogen *Candida albicans* were examined. Like budding yeast, *C. albicans* has a Kss1p-type pathway (Cek1p pathway [123]–[125]), and a p38-type pathway (CaHOG pathway [126]). The CaHOG MAPK CaHog1p is activated by osmotic stress (**Fig. 7A** [127]) and was also induced by tunicamycin, myriocin, and growth in galactose (**Fig. 7A**, 5 h at 30°C). Thus, the versatility of HOG pathway in sensing diverse stresses is conserved among several fungal species.

We also tested whether CaIre1p is required to mediate the HOG pathway response to galactose. The *ire1*Δ/*ire1*Δ double mutant was defective for producing the elevated levels of P∼CaHog1p seen during galactose treatment in wild-type cells (**Fig. 7B**). A strain lacking CaIre1p but containing a complemented version *ire1Δ/IRE1*, restored P∼CaHog1p activity (**Fig. 7B**). Thus, CaIre1p mediates the HOG pathway response to galactose.

Previous reports have shown that the *C. albicans* HOG pathway negatively regulates the Cek1p pathway [128]. In *C. albicans*, growth at high temperatures (37°C) is a potent inducer of dimorphism [129], [130]. Interestingly, growth of *C. albicans* cells in galactose induced P∼Cek1p levels only at 37°C in wild-type cells (**Fig. 7C**). The *C. albicans hog1*Δ/*hog1*Δ mutant grown in galactose showed elevated P∼Cek1p levels at 30°C and 37°C (**Fig. 7C**). Similarly, the *hog1*Δ/*hog1*Δ mutant showed hyper-invasive growth compared to wild-type cells (**Fig. 7D**). Thus, CaHog1p inhibits Cek1p pathway activity during growth in galactose.

The Cek1p pathway might also inhibit the CaHOG pathway. In the *cek1*Δ/*cek1*Δ mutant, elevated P∼CaHog1p levels were observed during growth in galactose at 30°C and 37°C (**Fig. 7E**). Therefore, the CaHog1p pathway is activated by galactose in a Ire1p-dependent manner. Under this condition, the CaHog1p pathway and Cek1p pathways are both activated and both modulate each other's activities. These results indicate that the signaling axis described in *S.cerevisiae* extends to the opportunistic pathogen *C. albicans*.

## Discussion

Differentiation into specialized cell types occurs during development and in response to extrinsic cues. Fungal species differentiate into the filamentous/hyphal cell type, which in pathogens occurs during colonization of the host. Using a genomic survey, RNA seq analysis, we identify a new role for a p38 MAPK pathway (HOG) in differentiation to the filamentous cell type in yeast. The HOG pathway is activated during growth in poor carbon sources through a regulatory circuit involving the AMPK Snf1p. Since deletion of *MIG1* alleviates the need for Snf1p, the regulation of Hog1p is likely to be downstream of Mig1p repressed genes. HOG pathway activation in this context also required the ER stress kinase Ire1p. The connection between Ire1p and the HOG pathway has been reported [63], [64]. Our study therefore connects AMPK and Ire1p in a regulatory circuit that governs p38 (**Fig. 7F**). The HOG pathway and the ERK-type filamentous growth pathway induced target genes to promote growth in galactose, but the pathways also modulated each other's activities. Such modulation optimized cell growth and morphogenesis to facilitate production of the filamentous cell type (**Fig. 7F**). Our findings therefore elucidate a signaling network that occurs during differentiation and highlights the critical role for pathway modulation in proper cell-type specification.

Nutrient sensing in yeast has been extensively studied. Well-established pathways mediate the response to carbon and nitrogen limitation. We show that limiting nutrients, like non-preferred carbon and nitrogen sources, activate the p38-type HOG pathway in yeast. As a result, glucose repression, the AMPK Snf1p, metabolic respiration, and the TCA cycle feed into HOG pathway signaling (**Fig. 7F**). The HOG pathway is well known for its ability to sense and respond to changes in external osmolarity. Multiple inducers activate the HOG pathway, including citric acid [131], hypoxia [132], cold stress [133] and defective sphingolipid biosynthesis [114]. Here we demonstrate a connection between nutrients and activation of the HOG pathway (**Fig. 7F**). In mammals, p38-type stress activated protein kinase (SAPK) pathways mediate AMPK-dependent metabolic reprogramming. The p38 pathway can alter the balance between survival and apoptosis [134]. p38 also regulates respiration in muscles, and gluconeogenesis in liver, and when mis-regulated can lead to problems that range from diabetes [135]–[137] to tumor malignancy [138].

We show that in yeast, metabolic respiration feeds into the HOG response and requires the UPR regulator Ire1p. Several inducers of the Ire1p have been identified [139] but to our knowledge, this is the first example of a connection between defects in metabolic respiration and Ire1p (**Fig. 7F**). Hints at this connection come from studies in mammalian cells. Ire1p can regulate AMPK function [140], and glucose levels are connected in some manner to Ire1p activity [141]. Elements of the ER stress pathway drive metabolic reprogramming in triple-negative breast cancer cells [142]. In tumor microenvironments, Ire1p is required to promote the balance between lipid and protein biosynthesis potentially at the level of ER production [143]. It is plausible that Ire1p regulates p38 activity in these settings as well. It is unlikely that the AMPK-Ire1p-p38 circuit governs all p38-type responses; however, the connections reported here may underlie nutrient-dependent p38-type responses in many settings.

Metabolic respiration (and defects in protein glycosylation) induce the HOG (p38) and filamentous growth (ERK) pathways. The two pathways do not depend on each other for activation, and they induce non-overlapping targets (herein and [23], [82]). Both pathways are redundant for full growth on galactose, and the two pathways modulate each other's activities. This complicated functional interplay between the pathways is critical for proper cell growth and optimal differentiation to the filamentous cell type. Generally speaking, p38 and ERK pathways can be activated in the same cells to together orchestrate complex responses that include cell differentiation [144], [145]. An important insight from our study is that p38 and ERK pathways modulate each other's activities to produce an optimal response. Such cross-wiring to fine tune responses underscores the importance of precision in cell differentiation.

## Materials and Methods

### Strains and Plasmids and Growth Conditions

Yeast and bacterial strains were grown by standard methods [146], [147]. Yeast strains were grown on YEP (yeast extract and peptone) medium containing 2% glucose (D) or 2% galactose (GAL) unless otherwise indicated. Cells were grown at 30°C. Strains are listed in Table 1. Primers used in the study are listed in Table 2. The plate-washing assay [148] and the single cell invasive growth assay [60] were performed as described. Biofilm assays were performed as described [120], except that galactose was used as a carbon source.

**Table 1:** Strains used in this study.

Strain	Genotype^b^	Reference
PC244^e^	*MATa ste4 lys2 GAL-STE4 his3::FUS1-HIS3 FUS1-lacZ pmi40-101*	[60]
PC313^a^	*MAT*a *ura3-52*	[161]
PC538	*MAT*a *ste4 FUS1-lacZ FUS1-HIS3 ura3-52*	[11]
PC560	*MAT*a *ste4 FUS1-lacZ FUS1-HIS3 ura3-52 snf1::KlURA3*	[60]
PC620	*MAT*a *ste4 FUS1-lacZ FUS1-HIS3 ura3-52 kss1::KlURA3*	This study
PC653	*MAT*a *ste4 FUS1-lacZ FUS1-HIS3 ura3-52 snf4::KlURA3*	[60]
PC1029	*MAT*a *ste4 FUS1-lacZ FUS1-HIS3 ura3-52 flo11::KanMX6*	[122]
PC1523	*MAT* **a** *ste4 FUS1-lacZ FUS1-HIS3 ura3-52 ssk1::NAT*	[16]
PC2053	*MAT*a *ste4 FUS1-lacZ FUS1-HIS3 ura3-52 pbs2::KanMX6*	[16]
PC2061	*MATa ste4 FUS1-HIS3 ura3-52 ssk1::NAT ste11::KlURA3*	[16]
PC2382	*MATa ste4 FUS1-HIS3 ura3-52 ste12::KanMX6*	[16]
PC3861	*MAT* **a** *ste4 FUS1-lacZ FUS1-HIS3 ura3-52 ste11::NAT*	[74]
PC3912	*MATa ste4 FUS1-HIS3 ura3-52 aco1::NAT*	Chavel et al, Submitted
PC4843	*MATa ste4 FUS1-HIS3 ura3-52 mig1::NAT*	[74]
PC4982	*MAT* **a** *ura3-52 ste7::HYG*	This study
PC5008^d^	*ura3::imm434/ura3::imm434 his1::hisG/his1::hisG hog1::loxP-ura3-loxP/hog1::loxP-HIS1-loxP CIp20 (URA3 HIS1)*	[127]
PC5035	*MATa ura3-52 pbs2::NAT*	[16]
PC6016^c^	YS11: *Mat a can1Δ::Ste2pr-spHIS5 lyp1Δ::Ste3pr-LEU2 his3::hisG leu2Δ0 ura3Δ0*	[160]
PC6031	*MATa ste4 FUS1-HIS3 ura3-52 ssk22::NAT ssk2::KlURA3*	This study
PC6032	*MATa ste4 FUS1-HIS3 ura3-52 ire1::KlURA3*	This study
PC6044^e^	*MATa ste4 lys2 GAL-STE4 his3::FUS1-HIS3 FUS1-lacZ pmi40-101 ire1::KlURA3*	This study
PC6047	*MAT* **a** *ura3-52 hog1::NAT*	This study
PC6048	*MAT* **a** *ura3-52 ire1::NAT*	Adhikari et al, Submitted
PC6050	*MATa ste4 FUS1-HIS3 ura3-52 elm1::HYG*	This study
PC6051	*MATa ste4 FUS1-HIS3 ura3-52 tos3::KlURA3*	This study
PC6052	*MATa ste4 FUS1-HIS3 ura3-52 tos3::KlURA3 sak1::NAT*	This study
PC6053	*MATa ste4 FUS1-HIS3 ura3-52 tos3::KlURA3 elm1::HYG*	This study
PC6076	*MATa ste4 FUS1-HIS3 ura3-52 mig1::NAT snf1::KlURA3*	This study
PC6083	*MATa ste4 FUS1-HIS3 ura3-52 tos3::HYG sak1::NAT elm1::KlURA3*	This study
PC6085	*MAT* **a** *ste4 ura3-52 FUS1-HIS3 ssk22::NAT*	This study
PC6086	*MATa ste4 FUS1-HIS3 ura3-52 ssk2::KlURA3*	This study
PC6087	*MATa ste4 FUS1-HIS3 ura3-52 sak1::NAT elm1::KlURA3*	This study
PC6088	*MATa ste4 FUS1-HIS3 ura3-52 sak1::NAT tos3::HYG*	This study
PC6111^d^	*CAF2-1 = URA3/ura3::imm434 IRO1/iro1::imm434*	[162]
PC6114^d^	*cek1Δ/Δ ura3/ura3 cek1Δ::hisG/cek1Δ::hisG*	[124]
PC6116^d^	CW34 WT	[163]
PC6144^d^	SF008P *ura3Δ::λimm434 arg4::hisG his1::hisG::pHIS1 ire1::UAU1 ura3Δ::λimm434 arg4::hisG his1::hisG ire1::URA3*	[163]
PC6145^d^	SF008P *ura3Δ::λimm434 arg4::hisG his1::hisG::pHIS1-IRE1 ire1::UAU1 ura3Δ::λimm434 arg4::hisG his1::hisG ire1::URA3*	[163]
PC6152	*MATa ste4 FUS1-HIS3 ura3-52 fum1::KlURA3*	This study
PC6153	*MATa ste4 FUS1-HIS3 ura3-52 mdh1::KlURA3*	This study
PC6154	*MATa ste4 FUS1-HIS3 ura3-52 idh1::KlURA3*	This study
PC6155	*MATa ste4 FUS1-HIS3 ura3-52 kgd1::KlURA3*	This study
PC6156	*MATa ste4 FUS1-HIS3 ura3-52 ptp2::KlURA3*	This study
PC6157	*MATa ste4 FUS1-HIS3 ura3-52 ptp3::NAT*	This study
PC6158	*MATa ste4 FUS1-HIS3 ura3-52 ptp2::KlURA3 ptp3::NAT*	This study
PC6272	*MAT* **a** *ura3-52 ste7::HYG pbs2::NAT*	This study

a All strains are in the Σ1278b background unless otherwise indicated.

b KlURA3 refers to the Kluyveromyces lactis URA3 cassette.

c Σ1287b deletion collection. The following mutants (*gal3Δ*, *gal4Δ*, *gal7Δ*, *gal10Δ*, *pmt1Δ*, *pmt2Δ*, *pmt3Δ*, *pmt4Δ*, *pmt5Δ*, and *pmt6Δ*) were used from this collection for this study.

d refers to the *Candida albicans* strains used in this study.

e refers to 246-1-1 strain background.

**Table 2:** Primers used for qPCR in the study.

Name	Sequence
*CTT1*-F	5′CGTACTCTGGTCATTCCTTCATC3′
*CTT1*-R	5′TGACAGTTCAGCAGCCTTATC3′
*HSP12*-F	5′TCTTGGTTGGGTCTTCTTCAC3′
*HSP12*-R	5′TCTTGGTTGGGTCTTCTTCAC3′
*DDR2*-F	5′TCATTTCTGCCATCTCTGTCTT3′
*DDR2*-R	5′ACTCCGGCGTTTAGTAGTTG3′
*STL1*-F	5′TGGGCATTAGGCCAGTTTATC3′
*STL1*-R	5′ATTGACCAGCAACCCTCTATTT3′
*ENA1*-F	5′GGGTCCTGTATGGCTTCATTTA3′
*ENA1*-R	5′GCCGCAGAACGTGATCTATAA3′
*GPD1*-F	5′TCTCCATCTGTGGTGCTTTG3′
*GPD1*-R	5′CTCACCCAAACCGACTCTTT3′
*RAX2*-F	5′ACCAATCGAGGACAGTGAATAG3′
*RAX2*-R	5′CGTATAAGCGCATTGGAAGATG3′
*RSR1*-F	5′GTTGCAGTTAAAGACGCAAGAA3′
*RSR1*-R	5′CGTATCCTGTTTCGCAGAACTA3′
*BUD8*-F	5′CACGGGACAGAACTCCATTATAG3′
*BUD8*-R	5′ATCGTGCCTGTCTTCTTTCC3′
*CLB1*-F	5′CCTTCTGCCGGAAACTCTATATT3′
*CLB1*-R	5′AGGCATGATGTACCAACCAG3′
*CLB2*-F	5′TGCGAATAATCCAGCCCTAAC3′
*CLB2*-R	5′GCTGTTGATCTTGATACGCTTTC3′
*SWE1*-F	5′TCTTCGGGCCTCGTATCTAA3′
*SWE1*-R	5′ATGGTCTCTTCCCTCCACTAA3′
*SHO1*-F	5′AACTACGATGGGAGACACTTTG3′
*SHO1*-R	5′TCGTAAGCATCATCGTCATCAG3′
*TEC1*-F	5′ATGTTTCCAGAAGCCGTAGTT3′
*TEC1*-R	5′TTTAGCACCCAGTCCAGTATTT3′
*WSC2*-F	5′AGCATGTGGACTTGGAAGAG3′
*WSC2*-R	5′CGAAGCAGACGGTGGAATAA3′
*STE12*-F	5′GCAATCTTACCCAAACGGAATG3′
*STE12*-R	5′AATCGTCCGCGCCATAAA3′

Plasmid YCp-Cdc12p-GFP was provided by J. Pringle [149], pFRE-lacZ by H. Madhani [150], pUPRE-lacZ by David Eide [151], and p8X-CRE-lacZ by H. Saito [73]. Plasmid selection was maintained in synthetic complete medium containing 2% glucose (SD) or 2% galactose (S-GAL) that lacked uracil (-URA) or leucine (-LEU). Gene disruptions were performed according to standard genetic techniques [152], [153].

### RNA Preparation

Total RNA was isolated by acid phenol method from 10 ml cultures of WT and *pbs2*Δ mutant grown in YEPD (5.5 hrs), YEP-GAL (5.5 hrs), YEPD+Tunicamycin (3 hrs) and YEPD+salt (10 min). Isolated RNA was purified over a RNAeasy column (Qiagen). RNA concentration was measured using the NanoDrop2000 spectrophotometer (Thermo Scientific, Wilmington, DE) and purity was established by the A_260_/A_280_ ratio. RNA was detected by running samples on an 8M Urea 6%polyacylamide gel, stained by ethidium bromide. Three independent inductions were evaluated for RNA-seq analysis. Total RNA integrity was checked using an Agilent 2200 TapeStation (Agilent Technologies, Inc., Santa Clara, CA) and quantified using a Trinean DropSense96 spectrophotometer (Caliper Life Sciences, Hopkinton, MA).

### RNA-seq Expression Analysis

RNA sequencing was performed at the Fred Hutchinson Cancer Research Center (Seattle, WA). RNA seq was performed in triplicate by sequencing RNA prepared from 3 different (independent) cultures. RNA-seq libraries were prepared from total RNA using the TruSeq RNA Sample Prep Kit (Illumina, Inc., San Diego, CA, USA). Library size distributions were validated using an Agilent 2200 TapeStation (Agilent Technologies, Santa Clara, CA, USA). Additional library QC, blending of pooled indexed libraries, and cluster optimization was performed using Life Technologies' Invitrogen Qubit 2.0 Fluorometer (Life Technologies-Invitrogen, Carlsbad, CA, USA). RNA-seq libraries were pooled (24-plex) and clustered onto a flow cell lane using an Illumina cBot. Sequencing was performed using an Illumina HiSeq 2500 in Rapid Mode employing a paired-end, 50 base read length (PE50) sequencing strategy.

Image analysis and base calling were performed using Illumina's Real Time Analysis v1.17 software, followed by ‘demultiplexing’ of indexed reads and generation of FASTQ files, using Illumina's CASAVA v1.8.2 software (http://www.illumina.com/software.ilmn). For analysis of the RNA seq data, reads of low quality were filtered out prior to alignment to the reference genome (*S. cerevisiae* assembly R64-1-1, Ensembl release 75) using TopHat v2.0.9 [154]. Counts were generated from TopHat alignments for each gene using the Python package HTSeq v0.5.4 (http://www-huber.embl.de/users/anders/HTSeq/doc/overview.html). Genes with low counts across all samples were removed, prior to identification of differentially expressed genes using the Bioconductor package edgeR v3.4.2 [155]. A false discovery rate (FDR) method was employed to correct for multiple testing [156]. Differential expression was defined as |log_2_ (ratio) |≥0.585 (±1.5-fold) with the FDR set to 5%.

### Quantitative RT-PCR Analysis

cDNA was synthesized using iScript cDNA synthesis kit (BioRAD, Carlsbad CA) according to manufacturer's protocol. PCR reactions were set-up using iQ SYBR Green Supermix (BioRAD, Carlsbad, CA). qPCR was performed using the following amplification cycles: initial denaturation for 8 min at 95°C, followed by 35 cycles (denaturation for 15 sec at 95°C and annealing for 1 min at 60°C). Expression of genes was quantified using the 2^−ΔΔCt^ method [157] where *ACT1* (actin) was used for normalization of expression values.

### Cell Inductions and Protein Immunoblot Analysis

To analyze HOG and filamentous growth pathway activity by phosphoblot analysis, cells were induced under the following conditions. For *S. cerevisiae*, cells were grown in YEPD to mid-log phase, and 0.4 M KCl was added for 5 min. For *C. albicans*, 0.4 M NaCl was used. For galactose, cells were grown in YEP-GAL medium to mid-log phase (5.5 hrs). For tunicamycin, cells were grown to mid-log phase in YEPD, and tunicamycin was added to cells for 3 hrs. [2.5 µg Sigma CAT. # T7765]. Antimycin [Sigma CAT # A8674] was added to mid-log phase cells for 3 hrs. (2.5 µg, 3 hrs), H_2_O_2_ [Sigma CAT #216763] was added to cells at a concentration of 5 mM for 20 min. Myriocin [Sigma CAT #M1177]. Yeast strains were grown in nitrogen free media (yeast nitrogen base without amino acids and without ammonium sulfate [1.7 g/L] BD Franklin Lakes, NJ; #233520) [31] supplemented with glucose as a carbon source. For phosphate free medium (yeast nitrogen base without amino acids and without phosphate [5.6 g/L], MP Biomedicals LLC, Solon, OH; #114027812) was supplemented with amino acids as a nitrogen source and glucose as a carbon source. For induction of *Candida albicans* pathways, strains were maintained at 30°C. Cells were grown to mid-log phase (∼5 hrs) in YEPD or YEP-GAL and treated with 0.5 M NaCl for 10 min, myriocin (5 mM for 10 min), and tunicamycin (2.5 µg for 3 hrs). The *pmi40-101* mutant was grown in YEPD medium supplemented with or without 50 mM mannose for 5 hrs. For strains that exhibited growth defects in galactose, input cell number (OD_600_) was increased to be equivalent to wild-type cells at mid log phase.

Cell extracts were prepared for immunoblot analysis according to established procedures [158]. Mid-log phase cells were harvested by centrifugation, and proteins were precipitated by trichloroacetic acid [TCA]. Cells were lysed in the TCA buffer (10 mM Tris HCl pH 8.0; 10%TCA; 25 mM ammonium acetate; 1 mM EDTA) containing glass beads using FastPrep-24 Instrument (MP Biomedicals LLC, Solon, OH). After high-speed centrifugation the pellet was thoroughly mixed in the resuspension buffer (0.1M Tris HCl pH 11.0; 3%SDS) and boiled for 5 min and centrifuged for 30 sec at 16000 g. To the supernatant, equal volume of 2× SDS loading dye (100 mM Tris HCl pH 6.8; 4%SDS; 0.2% Bromophenol Blue; 20% glycerol; 200 mM ß-mercaptoethanol) was added.

Protein samples were separated on 10% SDS polyacrylamide gels (SDS-PAGE) and transferred to nitrocellulose membranes (Protran BA85, VWR International Inc., Bridgeport NJ). The membrane was blocked in immunoblot buffer (5% nonfat dry milk, 10 mM Tris-HCl [pH 8], 150 mM NaCl and 0.05% Tween 20) for 16 h at 4°C. WesternBright MCF fluorescent Western blotting kit from Advansta Inc. (Menlo Park, CA; LPS #K-12045-D20) was used for detection. Pgk1p antibodies (Life Technologies, Camarillo, CA; #459250) were used as a loading control. P∼Hog1p was detected using phospho-p38 antibodies (Cell Signaling Technology, Danvers, MA; #9211). *S. cerevisiae* Hog1p was detected by Hog1p antibodies (Santa Cruz Biotechnology, Santa Cruz, CA; #yC-20). *C. albicans* Hog1p was detected by (Santa Cruz Biotechnology, Santa Cruz, CA; #y-215). Cdc2 p34 antibody that recognizes PSTAIRE motifs in cyclin dependent kinases was used as a loading control for Candida protein extracts (Santa Cruz Biotechnology, Santa Cruz, CA; #sc-53). Phosphorylated Kss1p was detected by p42/p44 antibodies (Cell Signaling Technology, Danvers, MA; #4370) and total Kss1p was detected by (Santa Cruz Biotechnology, Santa Cruz, CA; #6775). Secondary antibodies, goat α-mouse IgG–HRP (Bio-Rad Laboratories, Hercules, CA; #170-6516), goat α-rabbit IgG-HRP (Jackson ImmunoResearch Laboratories, Inc., West Grove, PA; #111-035-144), donkey α-goat IgG-HRP (Santa Cruz Biotechnology, Santa Cruz, CA; #sc-2020) were used and incubated for 1 hr at 20°C. Ponceau S (Sigma, St. Louis, MO; #P7170) was used to confirm equal loading among samples.

### ß-Galactosidase Assays

ß-galactosidase assays were performed as described [11]. Cells were grown in selective media (SD-URA) for 16 hrs and sub-cultured in YEPD or YEP-GAL media for 5.5 hrs. Three independent experiments were performed and the average values are represented. Error bars indicate the standard deviation between trials.

### Microscopy

Differential-interference-contrast (DIC) and fluorescence microscopy was performed with an Axioplan 2 fluorescent microscope (Zeiss) with a PLAN-APOCHROMAT 100×/1.4 (oil) objective (N.A. 0.17). Digital images were obtained with the Axiocam MRm camera (Zeiss). Image Acquisition and analysis was carried out using Axiovision 4.4 software (Zeiss).

### Bioinformatics Analysis

Heat maps were generated using MeV (MultiExperiment Viewer) (http://www.tm4.org/mev.html). ImageJ analysis was used to quantitate band intensity for protein gels and immunoblots (http://imagej.nih.gov [159]) using the invert function and by subtraction of background signals. SGD was used for yeast gene annotation and analysis (http://www.yeastgenome.org). RNA seq data was evaluated and represented by Microsoft EXCEL software.

## Supporting Information

Figure S1 The HOG and filamentous growth pathways. The HOG pathway (red) responds to osmotic stress. High osmolarity dampens Sln1p activity, thereby activating the ‘Sln1p’ branch. High osmolarity also stimulates the Sho1p branch, which is composed of proteins that are also required in the filamentous growth pathway (black). Several proteins regulate the filamentous growth pathway but not the HOG pathway (green). The filamentous growth pathway is induced by glucose limitation and glycosylation deficiency. Induction of each pathway by its respective inducer, orchestrates a different response.
(TIF)

Figure S2 Analysis of genes identified by comparative RNA seq analysis. A) Venn diagram showing genes repressed by salt, galactose, and/or tunicamycin. Numbers in parenthesis represent genes repressed by the ESR. B) Pie chart showing functional categorization of genes regulated by the HOG pathway in galactose. The ninety-five genes induced in galactose in a Pbs2-dependent manner (see Fig. 1B) were functionally classified by GO terms (www.yeastgenome.org) and represented by a pie chart in excel.
(TIF)

Figure S3 Role of different carbon sources and other nutrients in activation of the HOG and/or filamentous growth pathways. A) Wild-type cells (PC538) were grown in YEPD (GLU), YEP-GAL (GAL), YEP acetate (Acetate) and YEP ethanol+glycerol (Ethanol) for the times indicated. B) P∼Hog1p levels in response to the depletion of fixed nitrogen. Wild-type cells (PC538) were grown in SD+AA and SD-N (lacking nitrogen) medium for 5 hrs. C) Wild-type cells (PC538) were grown in YEPD (GLU), limiting glucose (0.2% GLU), YEP-GAL (GAL), or raffinose (RAF, 2%) to mid-log phase. D) Wild-type cells (PC538) were grown in SD+AA (+Phosphate) and SD−P (−Phosphate) medium for 5 hrs. E) P∼Hog1p levels in nitrogen-limiting media in mutants lacking the Sln1p- or Ste11p-branches of the HOG pathway. Wild-type (PC538), *ssk1*Δ (PC1523), *ssk2*Δ (PC6086), *ssk22*Δ (PC6085), *ssk2*Δ *ssk22*Δ (PC6031), *ste11*Δ (PC3861), *ste11*Δ *ssk1*Δ (PC2061), *pbs2*Δ (PC2053) and *hog1*Δ (PC6047) cells were grown in SD+AA and SD-N for 5 hrs.
(TIF)

Figure S4 Role of protein kinases that phosphorylate Snf1p in mediating HOG pathway activation in galactose. A) Wild-type cells (PC538) grown in YEPD or YEPD+ANT (5 µg) for 2.5 hrs 0.4M KCl was added to cells for 5 min. B) Wild type (PC538) and the *aco1*Δ (PC3912) mutant were grown in YEPD medium to mid-log phase and treated with 0.4M KCl for 5 min. C) P∼Hog1p levels in wild-type cells, the *snf1*Δ mutant (PC560) and the *snf4*Δ mutant (PC653) grown in YEP-GAL medium. D) Same cells grown in YEPD with 0.4M KCl for 5 minutes. E) P∼Hog1p levels in response to the depletion of fixed nitrogen. Wild-type cells (PC538), and the *snf1*Δ (PC560) and *snf4*Δ (PC653) mutants were grown in SD+AA and SD-N medium for 5 hrs.
(TIF)

Figure S5 Relationship between glycosylation defects and HOG pathway activation. A) P∼Hog1p levels in mutants defective for O-linked glycosylation. Wild-type (PC6016) cells and the *pmt1*Δ, *pmt2*Δ, *pmt3*Δ, *pmt4*Δ, *pmt5*Δ, and *pmt6*Δ mutants (obtained from the Σ1278b *MAT*a haploid deletion collection [160]) were grown in YEPD medium to mid-log phase. B) P∼Hog1p levels in response to tunicamycin treatment. Wild-type cells (PC538), and the *snf1*Δ (PC560) and *snf4*Δ mutants (PC653) were grown in YEPD medium for 3 hrs and then treated with or without 1 µg TUN for 3 hrs.
(TIF)

Figure S6 Analysis of the roles of the HOG and filamentous growth pathways in the response to growth in galactose. Activity of the cross-talk reporter (*ste4 FUS1-HIS3*) and invasive growth of strains lacking protein tyrosine phosphatases for the HOG pathway. Equal amounts of wild-type cells (PC538), the *ptp2*Δ (PC6156), *ptp3*Δ (PC6157), *ptp2*Δ *ptp3*Δ double mutant (PC6158), *pbs2*Δ (PC2053), and *ste12*Δ (PC2382) were spotted onto SD+AA, SD-HIS, SGAL+AA, SGAL-HIS, SD-HIS+3,4,5-amino-triazole (ATA), SGAL-HIS+ATA, and YEPD medium for 48 hrs. The plates were photographed, and the YEPD plates were washed in a stream of water and photographed again.
(TIF)

Table S1 Analysis of comparative RNA seq data for cells exposed to salt, galactose, or tunicamycin. Please note that there are multiple sheets in this table.
(XLSX)
