# Supplementary figures and images for: Metabolic Respiration Induces AMPK- and Ire1p-Dependent Activation of the p38-Type HOG MAPK Pathway
Source: PLoS Genet. 2014 Oct 30;10(10):e1004734. doi: 10.1371/journal.pgen.1004734 (PMC4214603; doi:10.1371/journal.pgen.1004734)

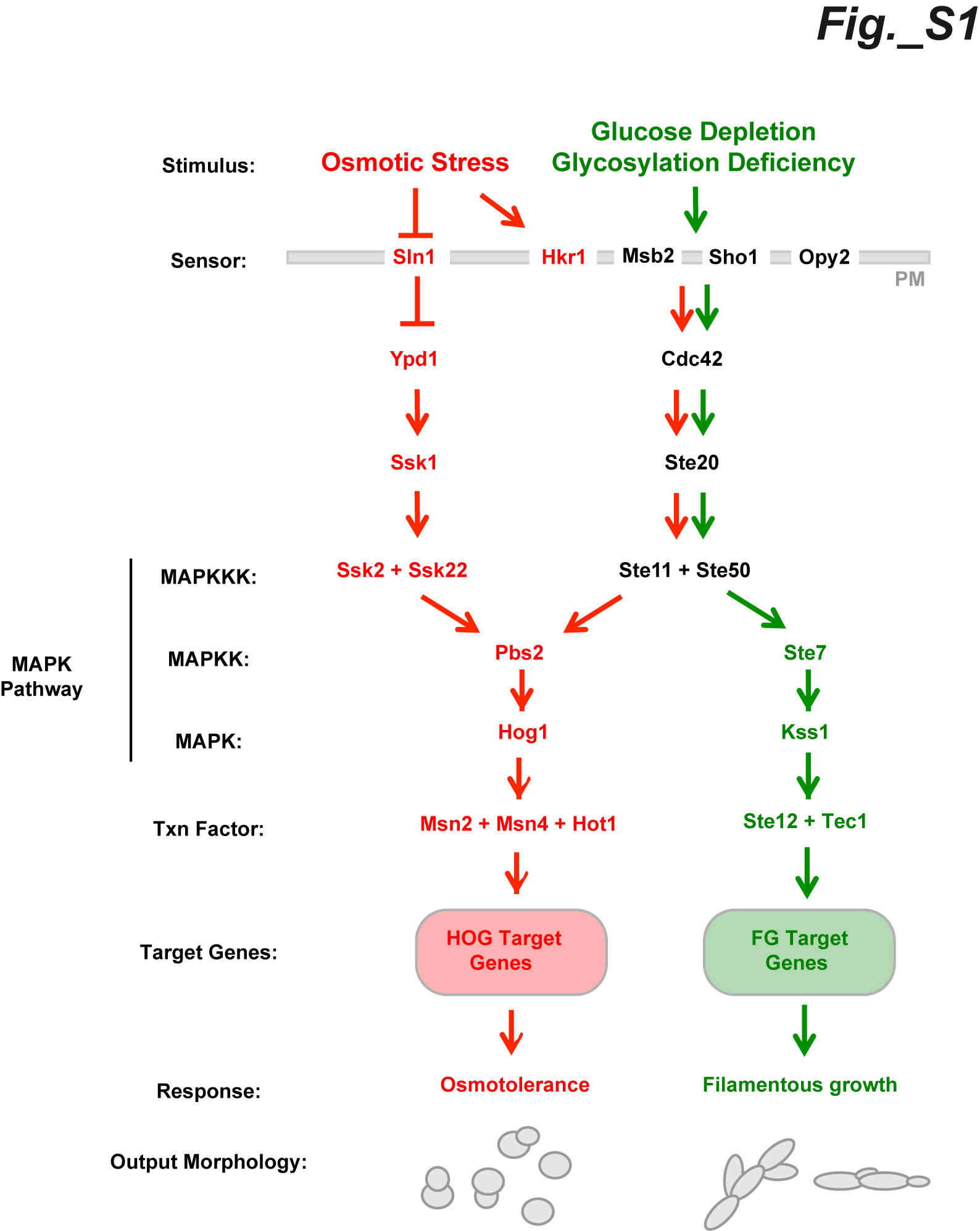

Supplement: Figure S1 — The HOG and filamentous growth pathways. The HOG pathway (red) responds to osmotic stress. High osmolarity dampens Sln1p activity, thereby activating the ‘Sln1p’ branch. High osmolarity also stimulates the Sho1p branch, which is composed of proteins that are also required in the filamentous growth pathway (black). Several proteins regulate the filamentous growth pathway but not the HOG pathway (green). The filamentous growth pathway is induced by glucose limitation and glycosylation deficiency. Induction of each pathway by its respective inducer, orchestrates a different response. (TIF) [file pgen.1004734.s001.tif]

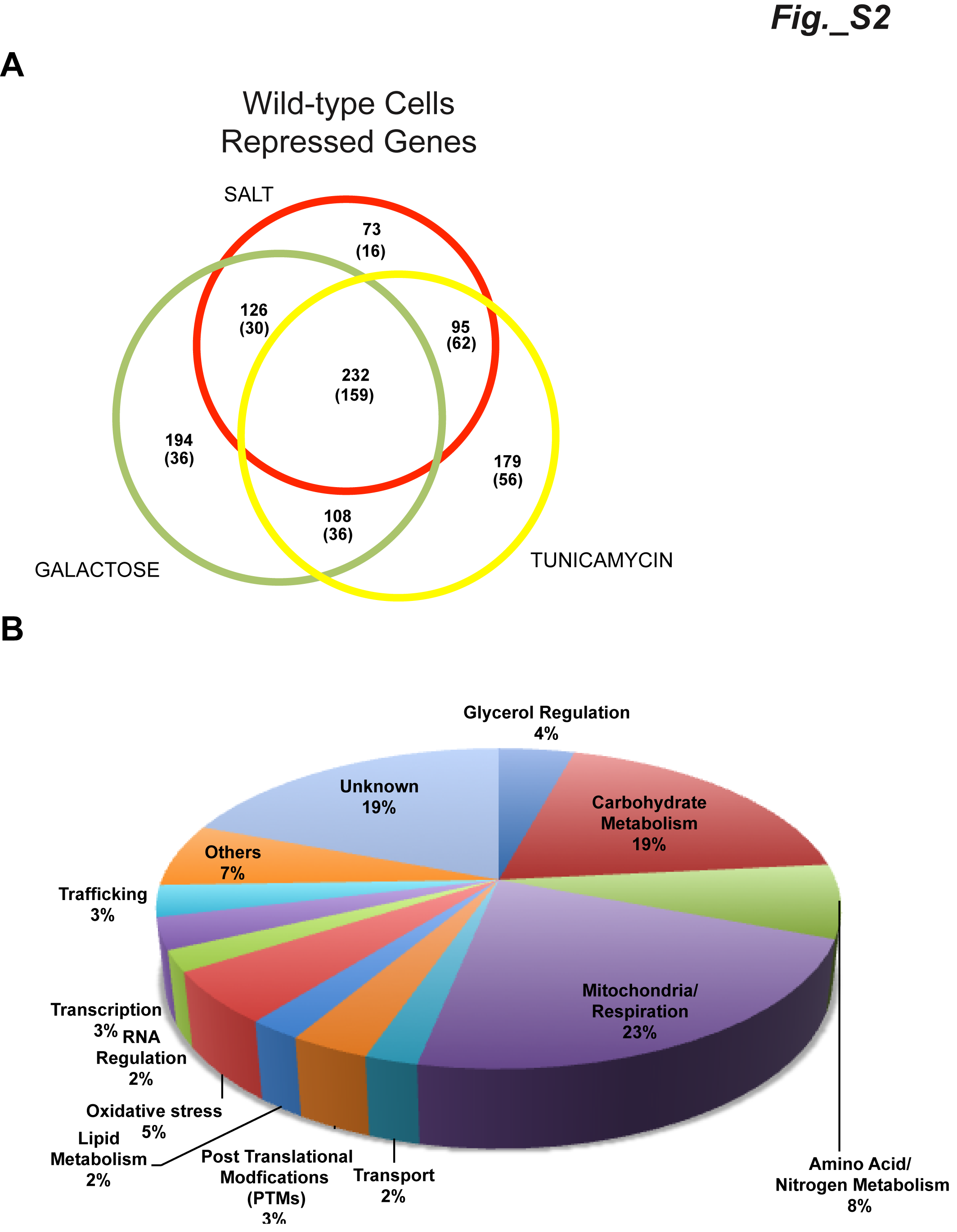

Supplement: Figure S2 — Analysis of genes identified by comparative RNA seq analysis. A) Venn diagram showing genes repressed by salt, galactose, and/or tunicamycin. Numbers in parenthesis represent genes repressed by the ESR. B) Pie chart showing functional categorization of genes regulated by the HOG pathway in galactose. The ninety-five genes induced in galactose in a Pbs2-dependent manner (see Fig. 1B) were functionally classified by GO terms (www.yeastgenome.org) and represented by a pie chart in excel. (TIF) [file pgen.1004734.s002.tif]

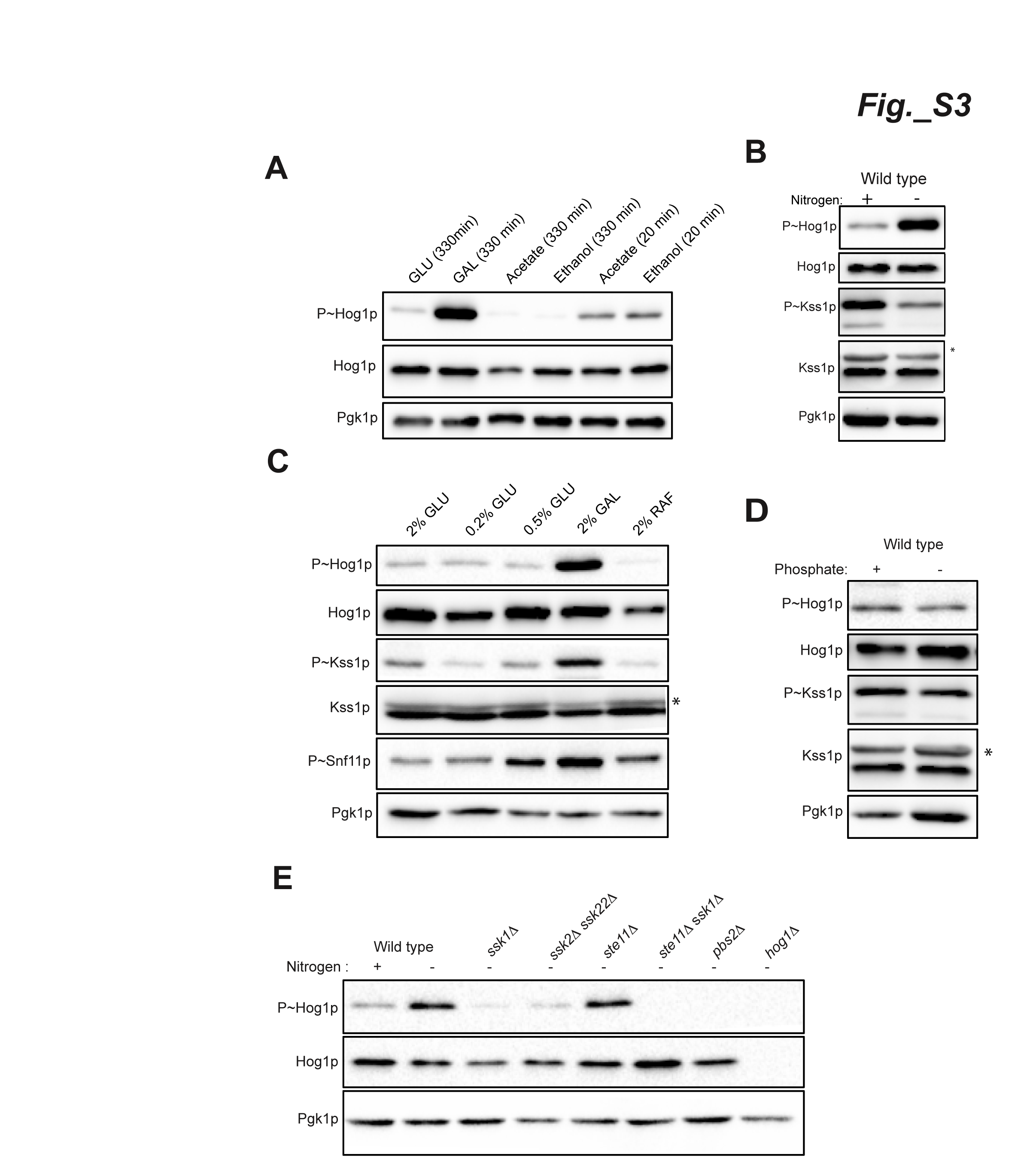

Supplement: Figure S3 — Role of different carbon sources and other nutrients in activation of the HOG and/or filamentous growth pathways. A) Wild-type cells (PC538) were grown in YEPD (GLU), YEP-GAL (GAL), YEP acetate (Acetate) and YEP ethanol+glycerol (Ethanol) for the times indicated. B) P∼Hog1p levels in response to the depletion of fixed nitrogen. Wild-type cells (PC538) were grown in SD+AA and SD-N (lacking nitrogen) medium for 5 hrs. C) Wild-type cells (PC538) were grown in YEPD (GLU), limiting glucose (0.2% GLU), YEP-GAL (GAL), or raffinose (RAF, 2%) to mid-log phase. D) Wild-type cells (PC538) were grown in SD+AA (+Phosphate) and SD−P (−Phosphate) medium for 5 hrs. E) P∼Hog1p levels in nitrogen-limiting media in mutants lacking the Sln1p- or Ste11p-branches of the HOG pathway. Wild-type (PC538), ssk1Δ (PC1523), ssk2Δ (PC6086), ssk22Δ (PC6085), ssk2Δ ssk22Δ (PC6031), ste11Δ (PC3861), ste11Δ ssk1Δ (PC2061), pbs2Δ (PC2053) and hog1Δ (PC6047) cells were grown in SD+AA and SD-N for 5 hrs. (TIF) [file pgen.1004734.s003.tif]

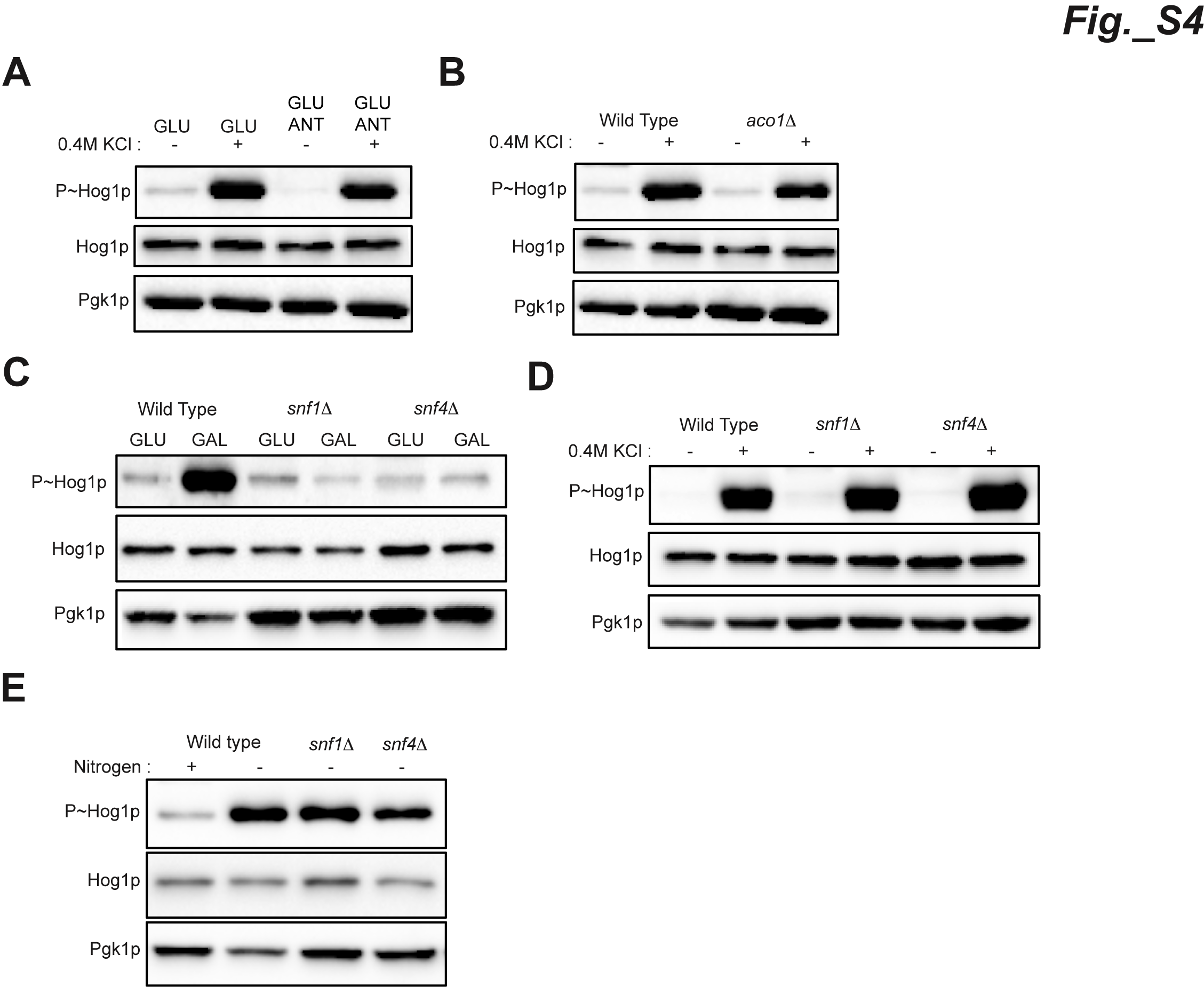

Supplement: Figure S4 — Role of protein kinases that phosphorylate Snf1p in mediating HOG pathway activation in galactose. A) Wild-type cells (PC538) grown in YEPD or YEPD+ANT (5 µg) for 2.5 hrs 0.4M KCl was added to cells for 5 min. B) Wild type (PC538) and the aco1Δ (PC3912) mutant were grown in YEPD medium to mid-log phase and treated with 0.4M KCl for 5 min. C) P∼Hog1p levels in wild-type cells, the snf1Δ mutant (PC560) and the snf4Δ mutant (PC653) grown in YEP-GAL medium. D) Same cells grown in YEPD with 0.4M KCl for 5 minutes. E) P∼Hog1p levels in response to the depletion of fixed nitrogen. Wild-type cells (PC538), and the snf1Δ (PC560) and snf4Δ (PC653) mutants were grown in SD+AA and SD-N medium for 5 hrs. (TIF) [file pgen.1004734.s004.tif]

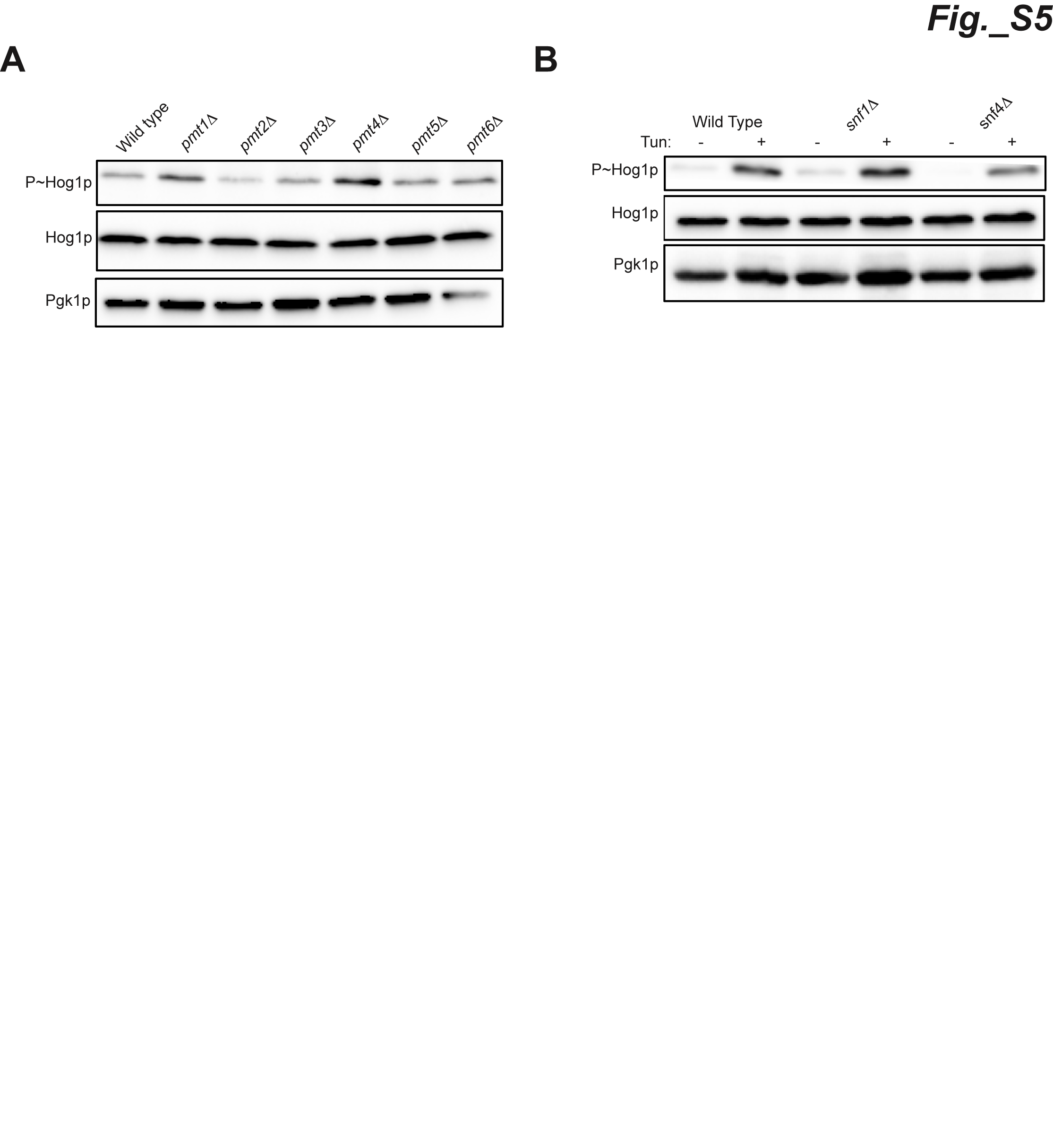

Supplement: Figure S5 — Relationship between glycosylation defects and HOG pathway activation. A) P∼Hog1p levels in mutants defective for O-linked glycosylation. Wild-type (PC6016) cells and the pmt1Δ, pmt2Δ, pmt3Δ, pmt4Δ, pmt5Δ, and pmt6Δ mutants (obtained from the Σ1278b MATa haploid deletion collection [160]) were grown in YEPD medium to mid-log phase. B) P∼Hog1p levels in response to tunicamycin treatment. Wild-type cells (PC538), and the snf1Δ (PC560) and snf4Δ mutants (PC653) were grown in YEPD medium for 3 hrs and then treated with or without 1 µg TUN for 3 hrs. (TIF) [file pgen.1004734.s005.tif]

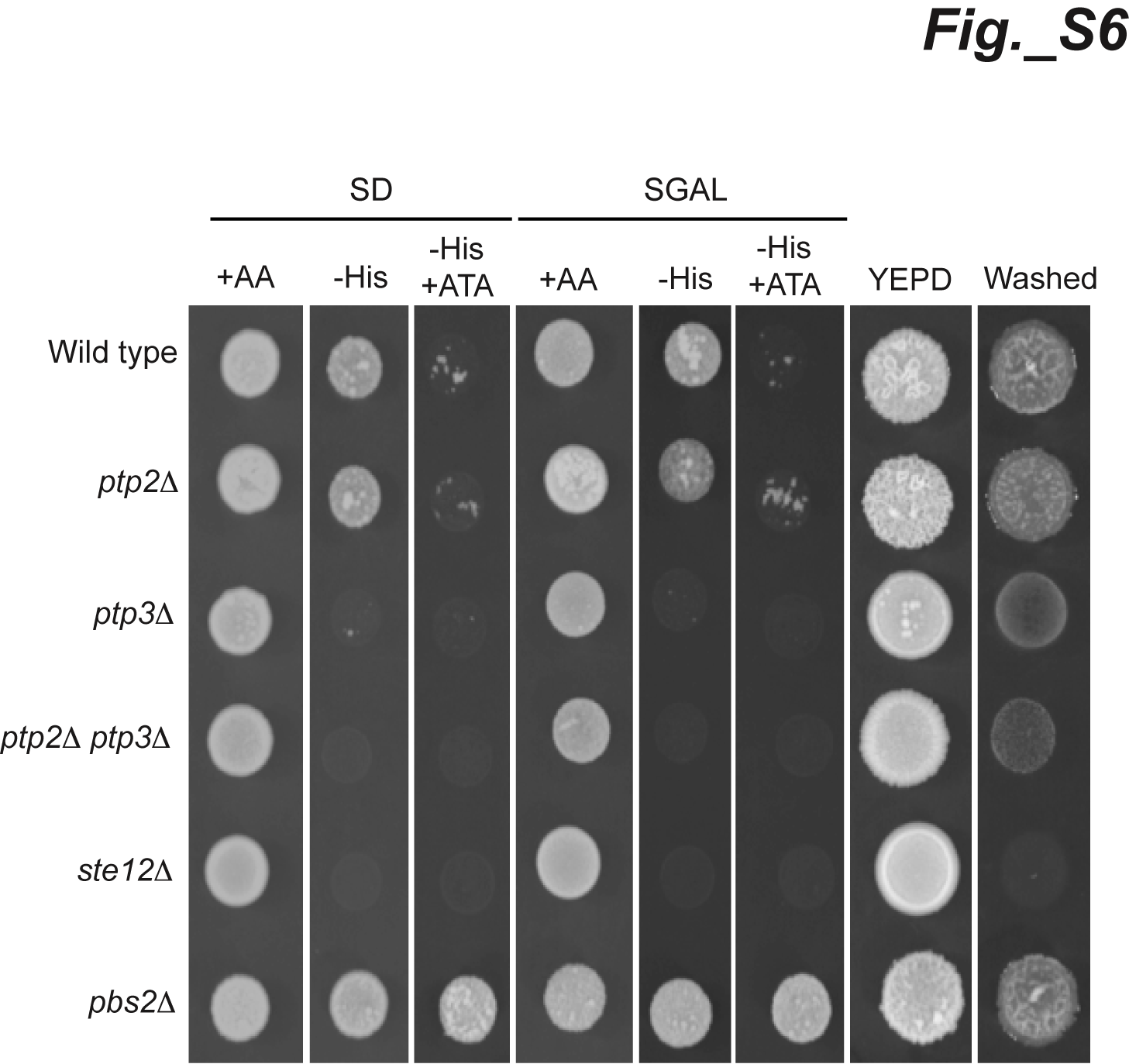

Supplement: Figure S6 — Analysis of the roles of the HOG and filamentous growth pathways in the response to growth in galactose. Activity of the cross-talk reporter (ste4 FUS1-HIS3) and invasive growth of strains lacking protein tyrosine phosphatases for the HOG pathway. Equal amounts of wild-type cells (PC538), the ptp2Δ (PC6156), ptp3Δ (PC6157), ptp2Δ ptp3Δ double mutant (PC6158), pbs2Δ (PC2053), and ste12Δ (PC2382) were spotted onto SD+AA, SD-HIS, SGAL+AA, SGAL-HIS, SD-HIS+3,4,5-amino-triazole (ATA), SGAL-HIS+ATA, and YEPD medium for 48 hrs. The plates were photographed, and the YEPD plates were washed in a stream of water and photographed again. (TIF) [file pgen.1004734.s006.tif]
